# Development of a prognostic model related to homologous recombination deficiency in glioma based on multiple machine learning

**DOI:** 10.3389/fimmu.2024.1452097

**Published:** 2024-10-07

**Authors:** Zhenyu Gong, Dairan Zhou, Haotian Shen, Chao Ma, Dejun Wu, Lijun Hou, Hongxiang Wang, Tao Xu

**Affiliations:** ^1^ Department of Neurosurgery, Changzheng Hospital, Naval Medical University, Shanghai, China; ^2^ Department of Neurosurgery, Klinikum rechts der Isar, Technical University of Munich, Munich, Germany; ^3^ Department of Neurosurgery, The Second Affiliated Hospital of Anhui Medical University, Hefei, Anhui, China; ^4^ Department of Neurosurgery, Changhai Hospital, Naval Medical University, Shanghai, China

**Keywords:** glioma, homologous recombination deficiency, prognosis, machine learning, risk model

## Abstract

**Background:**

Despite advances in neuro-oncology, treatments of glioma and tools for predicting the outcome of patients remain limited. The objective of this research is to construct a prognostic model for glioma using the Homologous Recombination Deficiency (HRD) score and validate its predictive capability for glioma.

**Methods:**

We consolidated glioma datasets from TCGA, various cancer types for pan-cancer HRD analysis, and two additional glioma RNAseq datasets from GEO and CGGA databases. HRD scores, mutation data, and other genomic indices were calculated. Using machine learning algorithms, we identified signature genes and constructed an HRD-related prognostic risk model. The model’s performance was validated across multiple cohorts. We also assessed immune infiltration and conducted molecular docking to identify potential therapeutic agents.

**Results:**

Our analysis established a correlation between higher HRD scores and genomic instability in gliomas. The model, based on machine learning algorithms, identified seven key genes, significantly predicting patient prognosis. Moreover, the HRD score prognostic model surpassed other models in terms of prediction efficacy across different cancers. Differential immune cell infiltration patterns were observed between HRD risk groups, with potential implications for immunotherapy. Molecular docking highlighted several compounds, notably Panobinostat, as promising for high-risk patients.

**Conclusions:**

The prognostic model based on the HRD score threshold and associated genes in glioma offers new insights into the genomic and immunological landscapes, potentially guiding therapeutic strategies. The differential immune profiles associated with HRD-risk groups could inform immunotherapeutic interventions, with our findings paving the way for personalized medicine in glioma treatment.

## 1 Introduction

Glioma represents a prevalent malignant neoplasm within the central nervous system (CNS). It represents around 30% of all cerebral tumors and up to 80% of malignant tumors (1). Among the various subtypes of gliomas, glioblastoma (GBM) stands out as the most virulent, characterized by its rapid progression and resistance to treatment, it accounted for the majority of gliomas (59.2%) (1). The 10-year survival rate for low-grade gliomas is 47%, but the median overall survival period for individuals with GBM is a mere 14-16 months, with a 5-year survival rate that does not exceed 6% (2, 3). Although significant advancements have been achieved in the realm of neuro-oncology, the treatment options available for gliomas, mostly consisting of surgical removal followed by adjunctive radiotherapy and chemotherapy, provide limited relief to patients, particularly those with high-grade gliomas (4). This situation underscores the exigency for innovative therapeutic interventions. The evolution of molecular biology has led to the identification of an array of molecular markers that could predict the survival rate of glioma patients, including genetic mutation status and DNA methylation. Consequently, the World Health Organization (WHO) implemented an updated classification system for CNS malignancies incorporating molecular diagnosis (5). However, the significant heterogeneity of gliomas leads to unavoidable tumor recurrence and medication resistance, highlighting the pressing necessity to identify novel biomarkers (6). These novel biomarkers could elucidate the pathological mechanisms of gliomas and aid in developing effective therapeutic strategies.

Homologous recombination (HR) is a process that conservatively repairs lethal DNA double-strand breaks (DSBs) with high precision (7). The BRCA1/2 genes are of significant importance in the homologous recombination repair pathway. When these genes have harmful mutations, they severely hinder the ability to repair DSBs, ultimately initiating HRD (8). In addition to BRCA1/2 mutations, HRD can be attributed to additional factors, including mutations in BARD1, PALB2, RAD51C, RAD51D, etc., as well as BRCA1 promoter methylation (9–11). This results in genomic instability, which gives rise to genomic scars, hence increasing one’s vulnerability to cancer (12), and facilitating the survival of detrimental tumors (13, 14). When exposed to PARP protein inhibition (PARPi), tumor cells exhibiting HRD, such as those with BRCA1/BRCA2 mutations in ovarian and breast malignancies, increase genomic fragility induced by disruption of single-strand break repair pathway (15, 16). Recent studies have shown that the development of resistance to temozolomide (TMZ) can be attributed to the amplification of homologous recombination in glioma cells, while its inhibition re-sensitizes the resistant cells, this indicates that HRD cells are especially sensitive to TMZ (17). HRD scores, which are derived from ‘genomic scars’, are designed to quantify HRD. It has been utilized in multiple types of cancers, such as breast, prostate, and ovarian cancer, showing its potential to progressively serve as a robust diagnostic marker in precision oncology (18–20). Nevertheless, as a result of the significant heterogeneity across various types of tumors, using varied thresholds for classification in different cancers might be more appropriate.

The field of machine learning (ML) has experienced significant advancements in recent years, offering an efficient and practical approach to analyzing vast and intricate datasets. Various machine-learning algorithms have been applied in glioma-associated clinical management, diagnosis, and classification aspects (21). For instance, identifying biomarkers for diagnosis and prognosis (22, 23), constructing recurrence prediction models for gliomas (24), developing models predicting the response to immunotherapy for gliomas (25), etc.

This study focused on investigating the significance of the HRD score in glioma, defining the HRD threshold, and identifying genes that are associated with HRD. Then, a multi-machine learning model based on HRD signature genes was constructed, it has the ability to accurately predict the prognosis of individuals with glioma. The prognostic ability of this model has been consistently demonstrated to be superior and stable across multiple datasets, as well as in comparison to currently published models. This model could also contribute to distinguishing immunological and genomic features and predicting the response to immunotherapy in glioma patients. Furthermore, utilizing the developed model, we identified prospective therapeutic agents for glioma and made predictions on their binding sites and affinity.

## 2 Materials and methods

### 2.1 Data download

To develop a glioma prediction model utilizing the origin of homologous recombination deficiency for precise clinical medicine in Gliomas, this study acquired the gliomas dataset in The Cancer Genome Atlas (TCGA-GBMLGG) from UCSC Xena (https://xena.ucsc.edu) (26). Patients’ gene expression sequencing data (n=1131) were downloaded, and the Count and FPKM values were standardized to TPM values. At the same time, the clinical data of patients were also downloaded, while the patients without clinical information were excluded. In the meantime, we acquired the TCGA database RNA-seq dataset and corresponding survival data for hepatocellular carcinoma (TCGA-LIHC), ovarian cancer (TCGA-OV), and osteosarcoma (GDC TARGET-OS) and bladder cancer immunotherapy dataset IMvigor210, these datasets were used for pan-cancer analysis of HRD. Then, HRD scores data that were collated from the pan-cancer atlas study (27) conducted by Thorsson et al. were downloaded. At the same time, the Mutation data of the patients was downloaded through the GDC, and the ‘Masked Somatic Mutation’ was chosen and visualized through the maftools R package to estimate each patient’s tumor mutation burden (TMB) (28). Fraction Genome Altered (FGA: measuring area of the chromosome copy number changes the percentage of the region), Mutation Count (mutations), and microsatellite instability (MSI) sensor score were acquired from the cBioPortal (http://www.cbioportal.org). In the end, a total of 641 samples that met the specified criteria were kept. GEO database (29) (https://www.ncbi.nlm.nih.gov/geo) was accessed to download two gliomas RNAseq data sets: GSE108474 (30) (Homo sapiens, GPL570, a total of 550 patients with tumor samples), GSE72951 (31) (Homo sapiens, GPL14951, a total of 112 patient tumor samples), and each dataset contained solid tumor samples of patients diagnosed with glioma. From CGGA (Chinese Glioma Genome Atlas) database (32) (http://www.cgga.org.cn/), the gene expression data and clinical information (such as survival time and status) of glioma patients were downloaded, with the data samples being sourced from Homo sapiens. All patients with pathological diagnoses of glioma were selected, and samples from patients without clinical stage information and survival information were excluded. Finally, two glioma patient datasets CGGA_693 (33) and CGGA_325 (34) with 970 tumor samples were retained for this study (**Supplementary Table S1**).

### 2.2 HRD score and neoantigen score calculation

The HRD score was determined by calculating the sum of three unweights components: loss of heterogeneity (LOH), telomeric allele imbalance (TAI), and large-scale state transition (LST) scores (35–37). The neoantigen load, defined as the number of peptides anticipated to attach to major histocompatibility complex (MHC) proteins, was calculated utilizing HLA types obtained from RNA sequencing data. The neoantigen burden is quantified through the enumeration of single nucleotide variants (SNVs) and insertion-deletion (Indel) mutations. Data pertaining to Homologous Recombination Deficiency (HRD) scores, the neoantigen load (encompassing both SNVs and Indels), as well as the mutation rate (represented by the count of single nucleotide mutations) were aggregated from the comprehensive pan-cancer Atlas investigation (27). by Thorsson et al. (**Supplementary Table S2**). The glioma patients within the top and bottom 20% brackets based on HRD scores were compared in terms of their FGA, Mutation Count, and MSI sensor scores. Subsequently, we identified the optimal threshold for the HRD score in relation to patient prognosis, which allowed us to categorize TCGA-GBMLGG patients into subgroups with high and low HRD scores.

### 2.3 Identify the signature genes associated with HRD score

The Limma R package was employed to analyze the difference between patients with high and low HRD scores, and log2fold change > 1 and P.adj < 0.05 were selected as cutoff values (38). The obtained log2foldchange greater than 1 was considered as the highly expressed genes in HRD, and the log2foldchange less than -1 was considered as the low-expressed genes in HRD. The volcano plot and differential ranking map served to illustrate the distribution of these genes, while the difference heat map was utilized to display the variations between the groups. Additionally, Spearman correlation analysis was employed to investigate the associations among the genes.

### 2.4 Multi-machine learning to achieve one-stop feature gene screening and prognostic model construction

To establish a robust prognostic model for Gliomas-HRD (1), we initially integrated 10 well-established algorithms, including Random Forest (RSF), Least Absolute Shrinkage and Selection Operator (LASSO), Gradient Boosting Machine, Survival Support Vector Machine (survival-SVM), Supervised Principal Component (SuperPC), Ridge Regression (Ridge), Cox Partial Least Squares Regression (plsRcox), CoxBoost, Stepwise Cox, and Elastic Network (Enet). Numerous studies have demonstrated the utility of machine-learning algorithms in developing robust prognostic models across various cancers (39–41). Notably, RSF, LASSO, CoxBoost, and Stepwise Cox possess capabilities for dimensionality reduction and variable selection. We leveraged these features to integrate them with other algorithms, resulting in a comprehensive suite of 76 machine-learning algorithm combinations (2). Subsequently, the TCGA-GBMLGG dataset was designated as the training cohort, and a set of 76 algorithms was utilized to identify crucial genes and develop a prognostic model using the previously identified feature genes (3). Finally, in the four test cohorts (CGGA-693, CGGA-325, GSE108474, GSE72951), we applied the features obtained from the training cohort to compute the Glioma-HRD risk score for each individual cohort. After evaluating the average C-index of the four test cohorts, we ultimately chose the most optimal consensus prognostic model for Gliomas-HRD. Following this selection, we proceeded to calculate its associated risk score, referred to as the HRD Score. The patients were categorized into high-risk and low-risk groups with HRD based on the median score value. The independent predictive value of this risk score was assessed using survival analysis and multivariate Cox analysis.

### 2.5 The performance of the HRD Score was compared with other signatures

In order to assess the prediction efficacy of the HRD Score, we conducted a comparison between the HRD Score and previously documented prognostic models for glioma biomarkers, including Tong_et.al, Cai_et.al, Tan_et.al, Zhang_et.al, Li_et.al. (42–46). In terms of glioma prediction performance, the predictive power of other biomarkers was compared with that of the HRD Score in four test cohorts (CGGA-693, CGGA-325, GSE108474, GSE72951). The codes and algorithms for the above five molecular markers were derived from their original studies.

### 2.6 Prognostic power of HRD score in pan-cancer

HRD plays a crucial role in cancer development. To investigate the consistent prognostic strength of the HRD Score across different types of tumors, we conducted a meta-analysis using three different datasets: liver hepatocellular carcinoma (TCGA-LIHC), ovarian cancer (TCGA-OV), and osteosarcoma (TARGET-OS). These analyses, based on the patient’s overall survival, were performed using the survival R package (https://CRAN.R-project.org/package=survival). To validate the prognostic significance of the HRD Score, Kaplan-Meier (K-M) curve analysis was utilized for survival assessment.

### 2.7 Gene set enrichment analysis enrichment analysis

We utilized the clusterProfiler R package to conduct GSEA. By adopting this method, we successfully derived normalized enrichment scores for individual gene sets, uncovering the signaling pathways enriched within groups exhibiting both high and low expression levels. (47). GSEA stands as a computational technique designed to assess whether a predefined set of genes exhibits a statistically significant difference between two distinct biological states. It is widely employed to infer alterations in the activity of pathways and biological processes across samples within expression datasets. For the purpose of GSEA analysis, we acquired the gene set “c2.cp.kegg.symbols.gmt” from the MSigDB database. This analysis was instrumental in evaluating the influence of the high-risk and low-risk groups on tumor-related pathways within the Kyoto Encyclopedia of Genes and Genomes (KEGG). FDR<0.25 was considered in the results.

### 2.8 Mutated gene oncogenic pathways, TMB, and MSI analysis

To investigate the single-nucleotide polymorphisms (SNPs) among different risk score categories in TCGA-GBMLGG patients, we engaged the maftools package. This facilitated the analysis of genes frequently mutated in patients belonging to both high- and low-risk groups. To evaluate the interactions between drugs and gene mutations, the drugInteractions function was used for analysis. The goal was to identify genetic mutations associated with susceptibility or resistance to specific drugs. In addition, biological oncogenic pathway analysis was performed on the mutation data to understand which biological oncogenic pathways are affected by gene mutations. We used the OncogenicPathways and PlotOncogenicPathways functions to achieve this goal. Meanwhile, we calculated glioma patient TMB data through maftools R package. Glioma patients with MSI - Sensor data is from the cBioportal database (https://www.cbioportal.org).

### 2.9 Immune infiltration and differential analysis of immunomodulators

CIBERSORT (accessible at https://cibersort.stanford.edu/), utilizes the principle of linear support vector regression to deconvolute the expression matrix of human immune cells into specific subtypes (48). In our investigation, we applied the CIBERSORT algorithm to evaluate the status of immune cell infiltration within the combined datasets of different glioma samples. Following this evaluation, the Wilcoxon test was utilized to examine the disparities in the infiltration of each immune cell type across various disease subgroups. P ≤ 0.05 was considered statistically significant. Furthermore, we conducted an assessment of the variance in expression of immune checkpoint (ICP) and immunogenic cell death (ICD) modulators between the high and low-risk groups, focusing specifically on ICP modulators such as PD-L1 and TIM-3, as well as ICD modulators including CALR and HMGB1. Subsequently, the ‘ESTIMATE’ R package was employed to conduct an analysis, comparing the tumor immune score, stromal score, and tumor purity across different groups.

### 2.10 Development and validation of potential therapeutic agents

The tumor immune dysfunction and exclusion (TIDE) is a methodology designed to simulate the mechanisms of tumor immune evasion, employed for the evaluation of potential responses to immune checkpoint blockade (ICB) therapy (49). TIDE predictions were conducted on the website http://tide.dfci.harvard.edu/, analyzing the differential percentage of immune therapy response predictions between groups. The IMvigor 210 immune therapy dataset was utilized to validate the reliability of the TIDE results. Following that, we used resources from the Cancer Treatment Response Portal (CTRP, available at https://portals.broadinstitute.org/ctrp/, encompassing 835 cancer cell lines (CCLs) and 481 compounds) and the Profiling Relative Inhibition Simultaneously in Mixtures (PRISM, which includes 1448 compounds tested in 482 cancer cell lines). These databases were utilized to identify potential therapeutic drugs specifically tailored for glioma patients categorized within the high-risk HRD group. Following the methodology outlined by Yang et al. (50) we undertook a multi-step process to identify potential drugs for glioma patients in the high-risk HRD group. (1) Initially, we acquired drug sensitivity data for cancer cell lines (CCLs) from the CTRP and PRISM repurposing datasets, as well as expression data for CCLs from the Encyclopedia of Cancer Cell Lines (CCLE) database. (2) It’s crucial to highlight that the CTRP and PRISM datasets furnish area under the curve (AUC) values, where lower AUC values signify increased sensitivity to the specific compound. (3) Utilizing the Wilcoxon rank-sum test, a differential analysis of drug responses between the high and low-risk groups based on their respective HRD Scores was conducted. A threshold of log2FC > 0.03 was established to pinpoint compounds that exhibited low AUC values, specifically within the high-risk HRD group. (4) Further refining our search, we employed Spearman’s correlation to screen for compounds demonstrating a negative correlation between AUC value and HRD Score, setting a threshold of R < -0.1. (5) The final step involved identifying potential drugs for patients in the high-risk HRD group. This was achieved by finding the intersection of compounds identified in steps (3) and (4).

The Connectivity Map (CMap; available at https://clue.io/) serves as a gene expression database developed to capture the variations in gene expression following the treatment of human cells with various compounds, including small molecules. It establishes a comprehensive repository linking compounds, changes in gene expression, and disease, serving as a crucial resource for biological applications in drug discovery and disease mechanism elucidation (51, 52). Using differential expression profiles, we applied CMap to identify potential glioma compounds, further validating our initial findings from the CTRP and PRISM databases.

### 2.11 Molecular docking

A comprehensive analysis of CTRP and PRISM combined with CMap was used to identify potential drugs with significant association with gliomas. We download the molecular structure of the drug from the PubChem database (https://pubchem.ncbi.nlm.nih.gov/). The molecular structures of target proteins of potential drugs were obtained from the Protein Data Bank (PDB) (http://www.rcsb.org/). Based on the CB - Dock2 (https://cadd.labshare.cn/cb-dock2/php/index.php) for the connection of potential drugs and targets with receptor visualization. PubChem is the largest global database for chemical information, allowing users to search for chemicals using names, formulas, structures, and other identifiers. The platform provides comprehensive information encompassing chemical properties, biological activities, safety and toxicity data, patent details, and references from scientific literature. PDB is currently the most important 2.5-dimensional (three-dimensional data expressed in a two-dimensional form) structure database of biological macromolecules (proteins, nucleic acids and sugars). This database archives three-dimensional structures of biological macromolecules—including proteins, polysaccharides, nucleic acids, and viruses—elucidated through experimental techniques such as X-ray single-crystal diffraction, nuclear magnetic resonance, and electron diffraction. CB-Dock2 is an improved version of the CB-Dock server for blind docking of protein ligands, which integrates cavity detection, docking, and homology template fitting into one. With the three-dimensional (3D) structure of proteins and ligands, we could predict their binding sites and affinities, enabling computer-assisted drug discovery.

### 2.12 Statistical analysis

In this study, we employed R software (version 4.3.1) to perform all statistical analyses. To compare two groups, the Wilcoxon rank-sum test was employed, whereas differences among more than two groups were assessed using the Kruskal-Wallis test. For correlation analyses, the Spearman correlation analysis was utilized. P < 0.05 was considered as the threshold of statistical significance.

## 3 Results

### 3.1 HRD score reflects the genomic instability of patients and can be used as a prognostic marker for glioma patients

**Figure 1:**
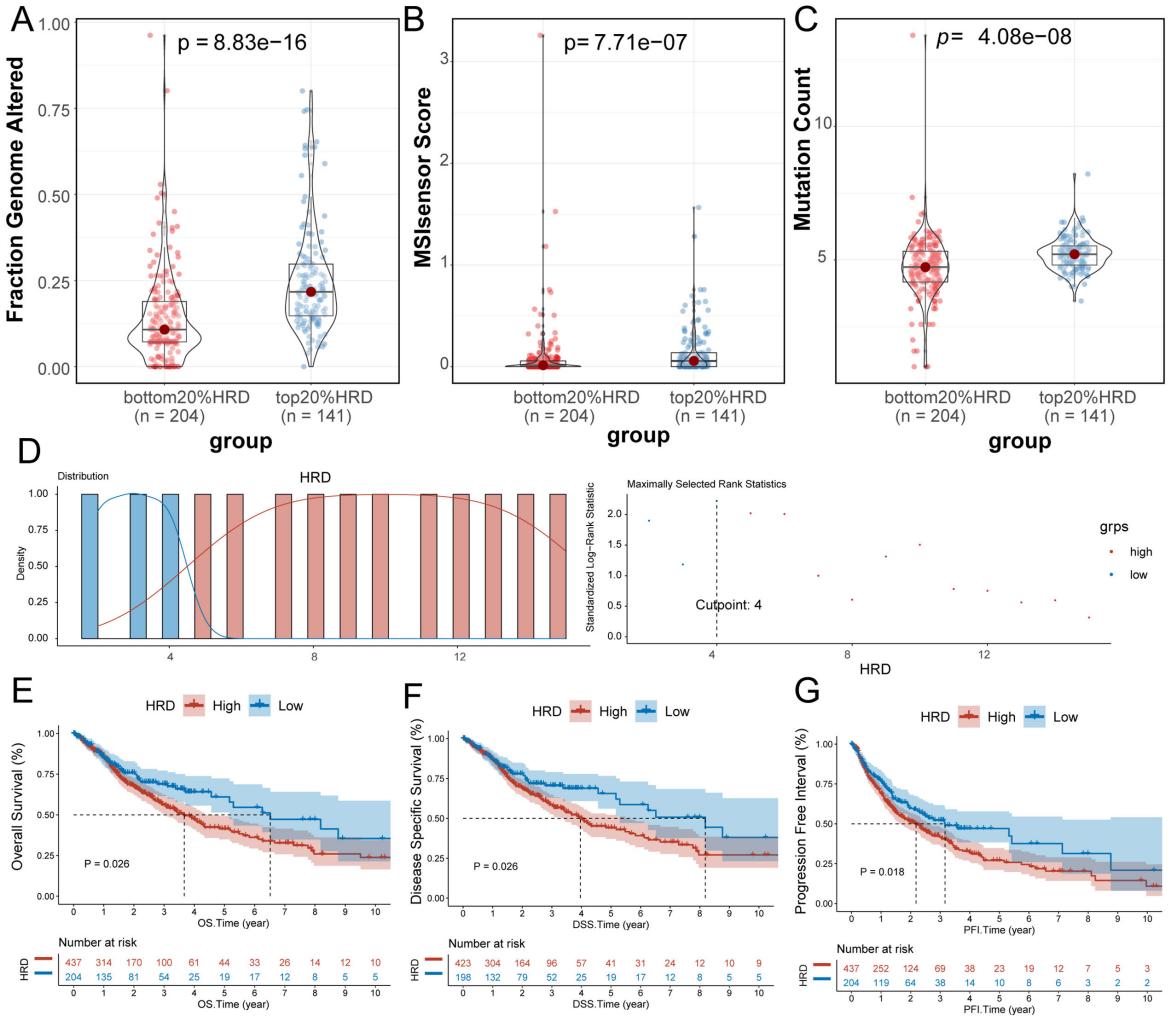
Significance of HRD score. **(A)** Violin plot of genomic alteration scores (Wilcoxon signed-rank test) for the top 20% of the HRD-score group and the bottom 20% of the HRD-score group; **(B)** violin plot of MSI in the top 20% group and the bottom 20% group of HRD score (Wilcoxon signed-rank test); **(C)** Violin plot of somatic mutations in the top 20% group of HRD score and the bottom 20% group of HRD score. (Wilcoxon signed-rank test); **(D)** Based on the prognosis of patients in the TCGA-GBMLGG cohort, the optimal cut-off value of HRD high and low score groups was divided; **(E)** K-M estimates of overall survival in patients with tumors grouped by HRD score in the TCGA-GBMLGG cohort; **(F)** K-M estimates of DSS according to HRD score in the TCGA-GBMLGG cohort for patients with tumors grouped by HRD score; **(G)** K-M estimates of PFI for patients with tumors grouped by HRD score and by HRD score in the TCGA-GBMLGG cohort. (HRD, Homologous recombination deficiency; K-M, Kaplan-Meier; TCGA, The Cancer Genome Atlas; GBM, Glioblastoma; LGG, Low-grade glioma; DSS, Disease-specific Survival; PFI, Progression-free Interval).

The HRD algorithm calculates the HRD score based on three foundational components: LOH, TAI and LST. To investigate the association between HRD score and other markers of genomic instability, including somatic mutation count, proportion of genomic alterations, and MSI, glioma patients were ranked in ascending order by HRD score, with those in the lowest 20% and the highest 20% selected. The proportion of genomic alterations (Wilcoxon signed-rank test, P=8.83e-16; **Figure 1A**), microsatellite instability (Wilcoxon signed-rank test, P=7.71e-07; **Figure 1B**) and somatic mutation counts (P=4.08e-08 by Wilcoxon signed-rank test, **Figure 1C**) were significantly higher in the group with the highest 20% of HRD scores. Furthermore, in the entire glioma cohort, we used the HRD score and prognostic information to find the best cutoff value to classify patients into the HRD high-expression group (HRD score > 4) and the HRD low-expression group (HRD score < 4) (**Figure 1D**). Survival analysis indicated that HRD score was a good prognostic indicator of Overall Survival (log-rank test, P=0.026, **Figure 1E**), Disease Specific Survival (log-rank test, P=0.026, **Figure 1E**). P=0.026, **Figure 1F**) and Progression-Free Interval (log-rank test, P=0.018, **Figure 1G**) both showed significant prognostic differences, with a significantly worse prognosis in the HRD high expression group.

### 3.2 Multi-machine learning models based on HRD signature genes

**Figure 2:**
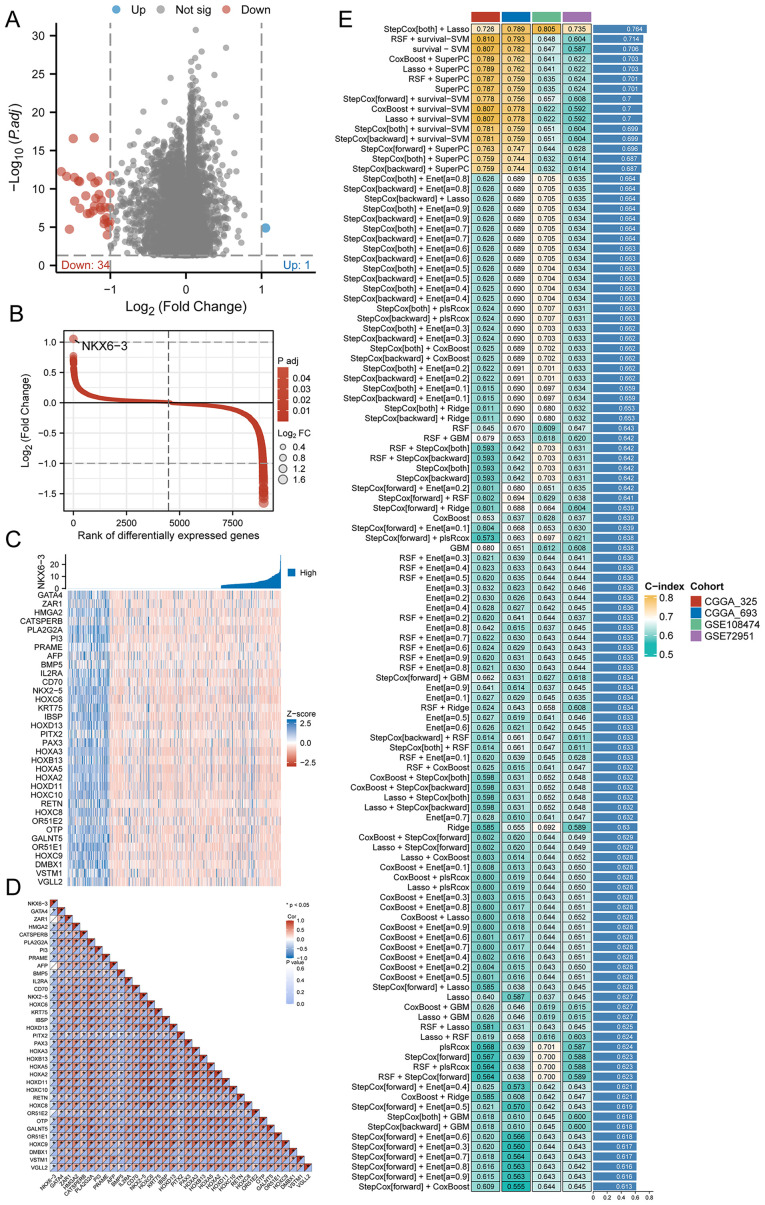
Selection of key genes and prognostic models. **(A)** Volcano map of differentially expressed genes between high and low HRD expression groups in the TCGA-GBMLGG dataset; **(B)** LogFC value distribution of differentially expressed genes in the high and low HRD expression groups in the TCGA-GBMLGG dataset; **(C)** Expression heatmap of 35 key genes between HRD high and low expression groups in TCGA-GBMLGG dataset; **(D)** Correlation heat map of 35 key genes between HRD high and low expression groups in TCGA-GBMLGG dataset; **(E)** C-index heatmap of 76 machine learning model combinations based on 35 key genes in validation sets CGGA-693, CGGA-325, GSE108474, and GSE72951. (HRD, Homologous recombination deficiency; TCGA, The Cancer Genome Atlas; GBM, Glioblastoma; LGG, Low-grade glioma; CGGA, Chinese Glioma Genome Atlas).

The identification of key HRD-related genes facilitates the development of glioma-predictive biomarkers to further identify HRD-derived gliomas. In order to identify key genes, we, for HRD to analyze differences between high and low expression groups, select P.adj < 0.05, | LogFC | > 1 differentially expressed genes as HRD-relevant features of the candidate. A total of 35 candidate genes, of which the down-regulated genes have 34, and the up-regulated gene has 1 (NKX6.3) (**Figure 2A**). The distribution of LogFC values for these 35 candidate genes is shown in **Figure 2B**. Compared with the up-regulated gene NKX6.3, the heatmap illustrating the differential expression of the remaining 34 genes displayed a clear difference in Z-scores between groups with high and low HRD expression (**Figure 2C**). Subsequently, we performed a correlation analysis of these 35 candidate genes, and the results indicated that most of the genes had significant correlations (**Figure 2D**). We then focused on the construction of the best prognostic model, as shown in **Figure 2E**, the StepCox[both] and Lasso combination with the highest mean C-index (0.764) among the 76 machine learning algorithms was selected as the final model.

### 3.3 Prognostic model establishment based on StepCox[both]+Lasso machine learning combination

**Figure 3:**
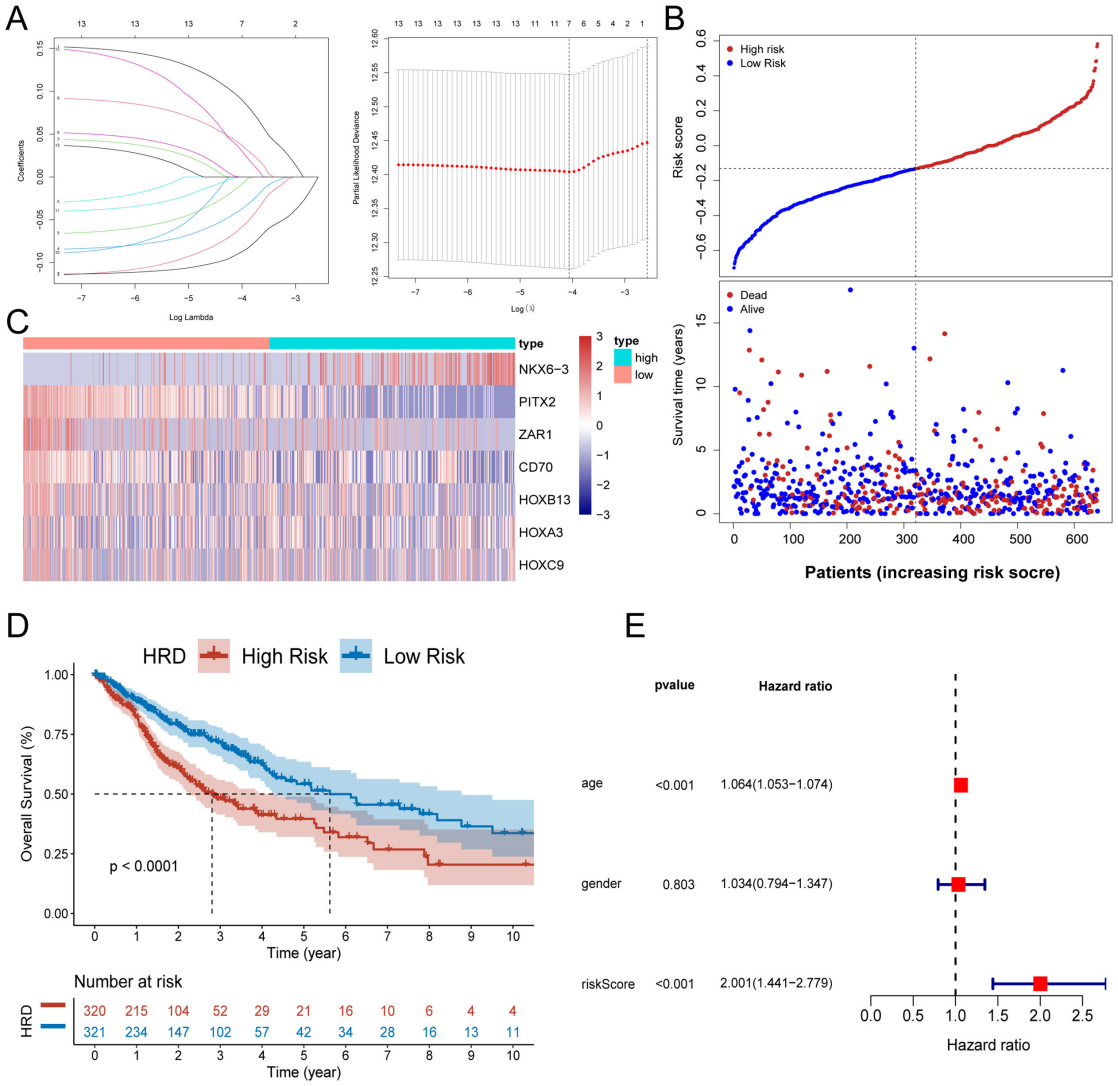
Development and validation of a risk model for HRD. **(A)** Lasso regression analysis showed that the number of variables corresponding to the best lambda value was 7. **(B)** Risk score distribution and survival status of glioma patients based on the Lasso model; **(C)** Heat map of characteristic gene expression based on the Lasso model; **(D)** The KM curve of high and low-risk patients in the training set TCGA-GBMLGG showed that the prognosis of patients in the high-risk group was significantly worse; **(E)** Forest plot of independent prognostic value of HRD risk score evaluated by multivariate Cox regression analysis. (K-M, Kaplan-Meier; TCGA, The Cancer Genome Atlas; GBM, Glioblastoma; LGG, Low-grade glioma).

**Figure 4:**
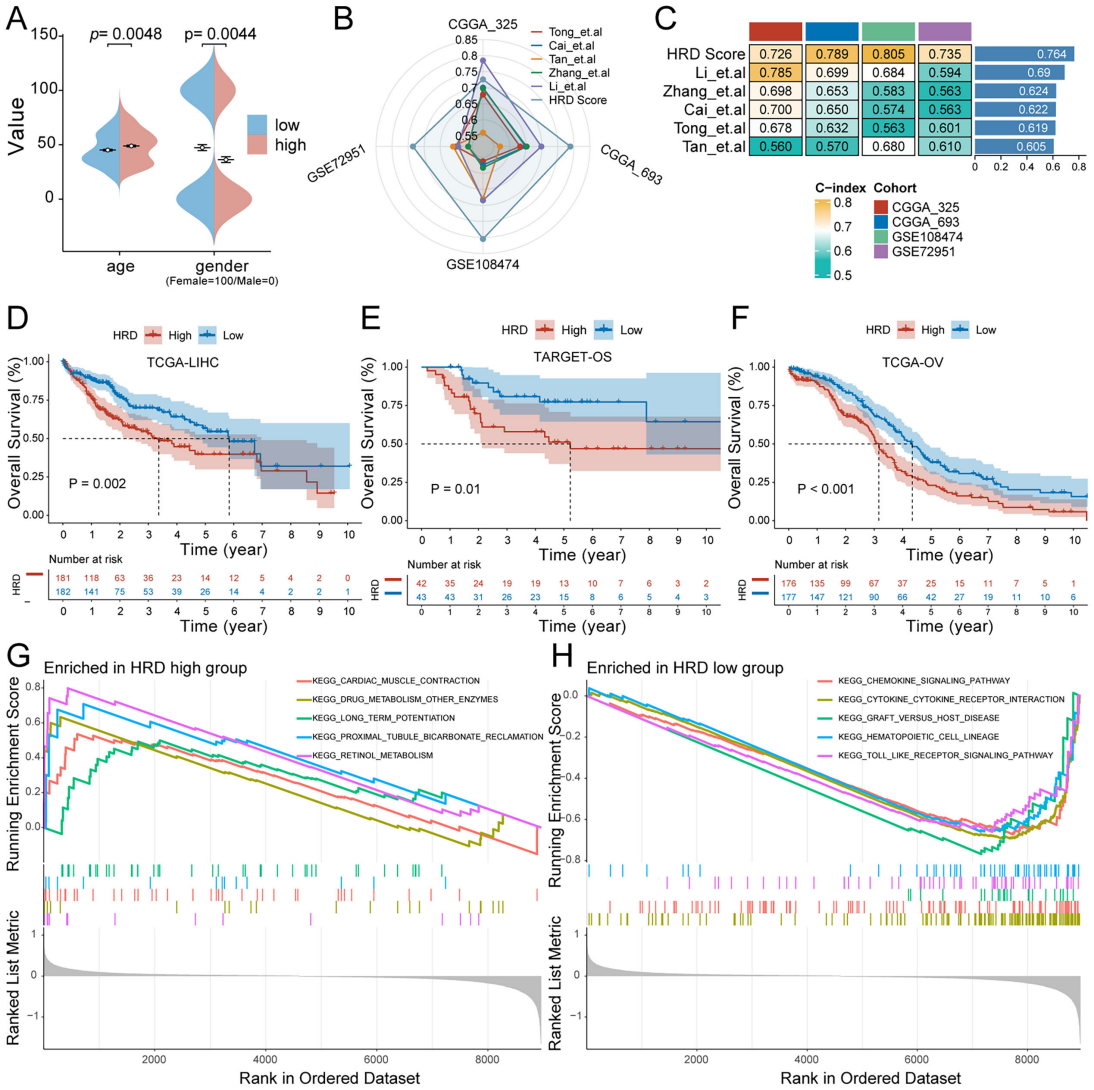
Characteristics of the HRD risk score. **(A)** Age and sex differences between HRD high and low-risk groups; **(B)** Radar plot of C-index distribution of HRD-Score prognostic prediction model and other published prognostic prediction models; **(C)** HRD Score and C-index heat map of other published prognostic prediction models; **(D)** The KM curve of the hepatocellular carcinoma dataset TCGA-LIHC showed that the prognosis of patients in the high-risk group was significantly worse (P=0.002). **(E)** The KM curve of Target-OS high and low-risk patients showed that the prognosis of the high-risk group was significantly worse (P=0.01). **(F)** The KM curve of the high - and low-risk patients in the ovarian cancer dataset TCGA-OV showed that the prognosis of the high-risk group was significantly worse (P<0.001). **(G)** HRD high-risk group pathway enrichment; **(H)** HRD low-risk group pathway enrichment. (HRD, Homologous recombination deficiency; KM, Kaplan-Meier).

According to the C-index comparison of the above 76 machine learning combination models, we selected the combination of the optimal model StepCox[both] and the Lasso algorithm to construct the HRD-related prognostic prediction model for glioma patients. The 35 key genes were further screened by the StepCox[both] algorithms, and the screening results were used to construct the Lasso prognostic model. We used Lasso analysis to reduce the dimension (**Figure 3A**), and multivariate Cox regression analysis to finally select and obtain 7 key genes with the best model (**Figure 3B**). The median expression of these 7 key genes was used as the cut-off value to divide the subgroups, and we performed prognostic analysis on them respectively. The respective K-M survival curves are shown in **Supplementary Figures S2A–G**, among which NKX6.3, PITX2, and CD70 genes showed significant prognostic differences. Based on the penalty coefficients of important signature genes calculated by multivariate Cox analysis (**Supplementary Table S3**), the gene expression levels were multiplied by their respective coefficients and subsequently summed up to derive a risk score, which was calculated for every individual sample. On the basis of the patient’s risk score and gene expression values, we generated a risk-factor heat map (**Figure 3C**). The K-M curve showed that patients in the high-risk group had a significantly worse prognosis, and there was a significant difference in survival between the high and low-risk groups (log-rank p <0.0001) (**Figure 3D**), which was validated in three additional datasets (**Supplementary Figures S1A–C**). Subsequently, we evaluated the independent prognostic power of the HRD risk score combined with other clinical information. Multivariate Cox regression analysis indicated that, similar to patient age, the HRD risk score could be used as an independent predictor of the prognosis of glioma patients. Additionally, we observed significant differences in age and gender characteristics when comparing the high-risk and low-risk groups categorized based on their risk scores. Specifically, the high-risk group tended to comprise elderly male patients (**Figure 4A**).

### 3.4 Comparison of HRD score with other prognostic models and evaluation of generalization ability

To validate the prognostic power of the HRD Score model, the predictive performance of the HRD Score was compared with that of currently published prognostic prediction models for gliomas. The results showed that the predictive performance of the HRD Score was high and stable in the validation sets CGGA-693, CGGA-325, GSE108474, and GSE72951 (**Figure 4B**). The average C-index of the model was significantly better than that of the other models (**Figure 4C**). The phenomenon of HRD has a significant effect on tumor progression not only in glioma but also in other tumor types. To explore whether the prognostic value of HRD Score can be generalized, we further evaluated the prognostic performance of HRD Score in TCGA-LIHC (hepatocellular carcinoma), TCGA-OV (Ovarian cancer), and TARGET-OS (osteosarcoma) datasets. The results of the KM survival curve showed that the HRD Score had a significant prognostic difference not only in glioma patients but also in HCC (P=0.002), OS (P=0.01), and EOC (P<0.001) patients.

### 3.5 Pathway enrichment and genomic features comparison between HRD high-risk and low-risk groups

**Figure 5:**
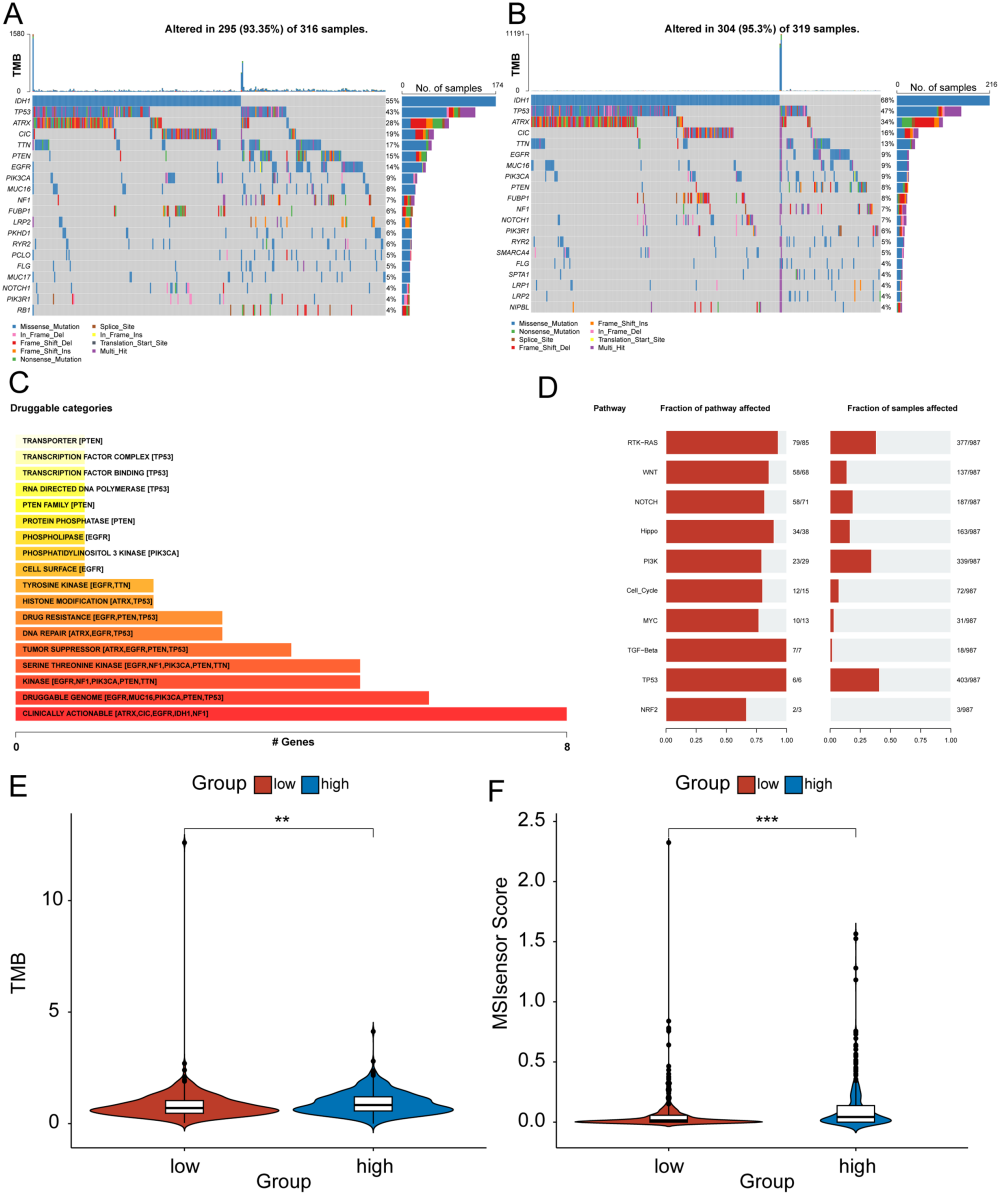
Genomic differences between high - and low-risk rroups for HRD. **(A)** Mutational profiles of common tumorigenic driver genes among patients in the high-risk group. The mutation information of each gene in each sample is shown in the waterfall plot, and various colors indicate different mutation types. The bar above the legend shows the mutation load; **(B)** Mutational profiles of common tumorigenic drivers among patients in the low-risk group. The mutation information of each gene in each sample is displayed in the waterfall plot, and various colors indicate different mutation types. The bar above the legend shows the mutation load; **(C)** The enrichment bar chart of the mutant gene patent drug pathway based on the mutation data of glioma patients. The color depth and the length of the bar chart represent the number of mutant genes enriched in this pathway. For pathways with more than 5 mutant genes enriched, only the top five mutant genes are shown; **(D)** Oncogenic pathway enrichment bar chart based on mutation data of glioma patients, the left side shows the proportion of pathways affected by mutant genes in a specific pathway, and the right side shows the proportion of affected patient samples; **(E)** Violin plot of the difference in tumor mutation burden between high and low-risk groups; **(F)** Violin plot of the difference in MSI between high and low-risk groups. (MSI, Microsatellite instability).

To investigate the difference in signal pathway enrichment between the high and low-risk groups of HRD, GSEA was performed. The results indicated that the top five gene pathways significantly enriched in the high-risk group of HRD were cardiac muscle contraction, drug metabolism, other enzymes, long-term potentiation, proximal tubule bicarbonate induction, and retinol metabolism (**Figure 4G**). The top five gene pathways significantly enriched in the low-risk group of HRD were the chemokine signaling pathway, cytokine cytokine receptor interaction, graft versus host disease, hematopoietic cell lineage, and toll-like receptor signaling pathway (**Figure 4H**). We then further evaluated the impact of HRD risk score on the changes in the level of genetic variants, including SNPS and copy number variations (CNVs), in glioma patients. The results of single nucleotide mutation analysis of common driver genes during tumorigenesis showed that the genes with high mutation levels were similar or close between patients with high scores of the HRD-related model and patients with low scores. However, the proportion of gene mutations remained predominantly higher in the high-risk group, particularly noticeable within the top five most frequently mutated genes (**Figures 5A, B**). Based on the glioma patient’s mutation data, we identified gene mutations associated with specific drug sensitivity or resistance, showing that glioma patient mutant genes are closely linked to multiple proprietary drug pathways (**Figure 5C**). In addition, we identified oncogenic pathway enrichment of mutated genes in glioma patients, with the highest enrichment in the RTK-RAS signaling pathway, an important cell signaling pathway. It holds a crucial function in various biological processes, including cell growth, differentiation, survival, and proliferation (**Figure 5D**). A differential analysis of the differential TMB (**Figure 5E**) and the MSI sensor score (**Figure 5F**) that characterized genomic instability among the patients in the high and low-risk groups showed that the corresponding metrics were notably elevated in the high-risk group, indicating a more pronounced genomic instability in the high-risk group. Furthermore, significant statistical differences were found in both mutation count (**Supplementary Figure S2H**) and genomic alteration score (**Supplementary Figure S2I**) between the two groups. The high-risk group exhibited higher values in terms of mutation count and genomic alteration score, underscoring the genomic instability characteristic of the high-risk HRD group.

### 3.6 Analysis of differences in immune infiltration and immunomodulators between high and low-risk groups of HRD

**Figure 6:**
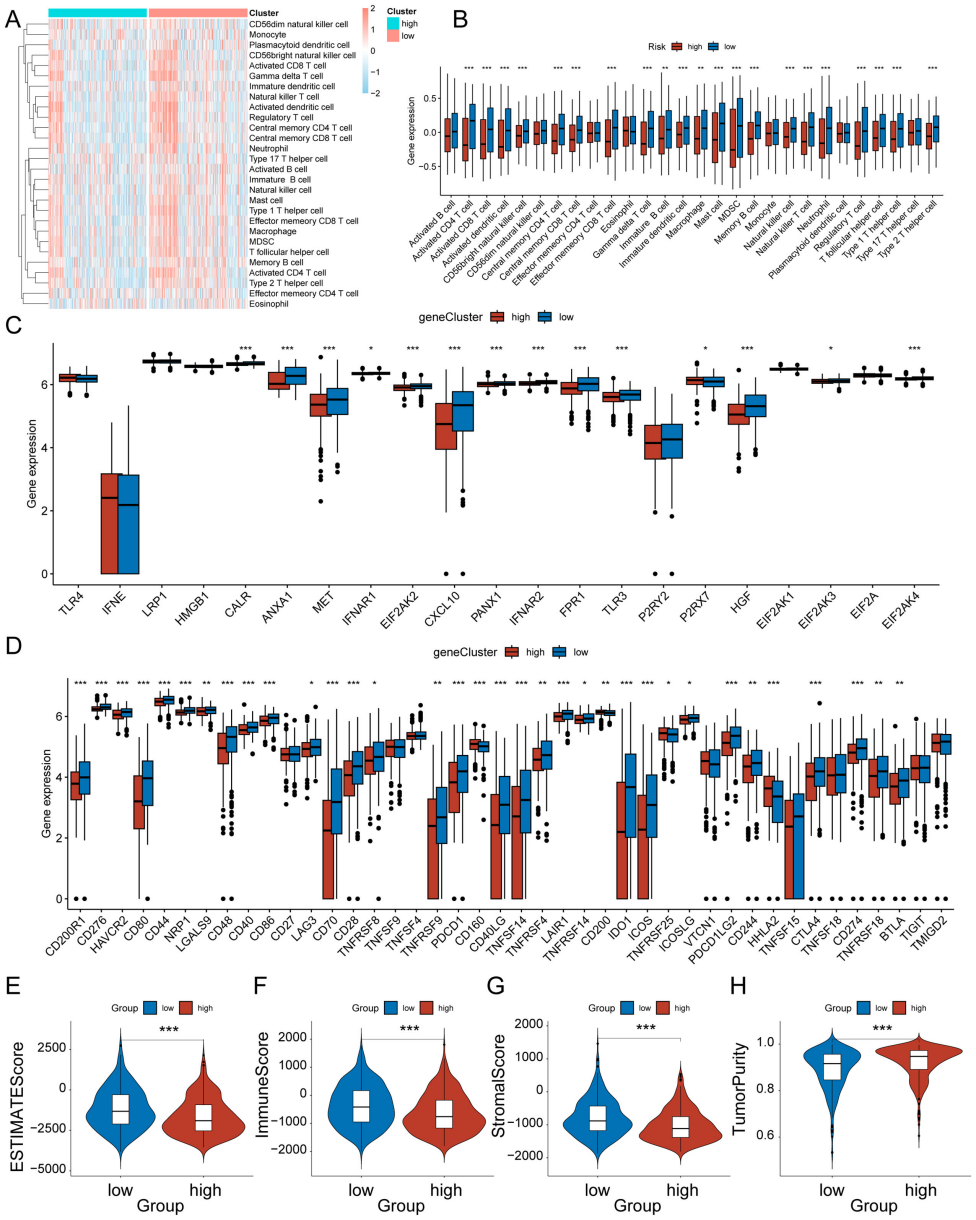
Characteristics of the immune microenvironment between high-risk and low-risk groups of HRD. **(A)** Heat map of infiltration levels of 28 immune cells between HRD high and low-risk groups. **(B)** Box plot of the differences in infiltration levels of 28 immune cells between HRD high and low-risk groups; **(C)** Box plot of differences in ICD modulator expression levels between HRD high and low-risk groups; **(D)** Box plot of the differences in expression levels of ICP modulators between HRD high and low-risk groups; **(E)** Violin plot of ESTIMATE score differences; **(F)** Violin plot of the differences in immune scores; **(G)** Violin plot of matrix score difference; **(H)** Violin plot of the difference in tumor purity. * represents p < 0.05, ** represents p < 0.01, and **** represents p < 0.001.

Immune cells are important in the development and progression of glioma. We evaluated the status of 28 immune cell distributions by ssGSEA and compared the abundance of different immune cell distributions by Wilcoxon to evaluate the difference in immune cell infiltration between the high and low-risk groups of HRD, which was more pronounced in the low-risk group (**Figure 6A**). **Figure 6B** shows the distribution differences of different immune cells between the high and low risk groups of HRD. In general, the low-risk group of HRD had a higher level of immune infiltration. Previous research has demonstrated that ICPs and ICD modulators significantly influence host anti-tumor immunity, impacting the effectiveness of mRNA vaccines in turn. And there is an important link between homologous recombination deficiency and immune regulation. Therefore, we evaluated the differential expression of ICP and ICD modulators in the HRD high and low-risk groups. Most ICD modulators were statistically significant upregulated in the low-risk group (**Figure 6C**), and among ICP modulators, the low-risk group remained statistically significant upregulated (**Figure 6D**). This suggests that patients in the low-risk group could potentially respond better to immunotherapy, while those in the high-risk group may tend to have immune-tolerant subtypes. ESTIMATE analysis was performed to explore the relationship between the tumor immune score, stroma score and tumor purity in two groups. The ESTIMATE score was significantly higher in the low-risk group of HRD (**Figure 6D**), with both the immune score (**Figure 6E**) and the stroma score (**Figure 6F**) being higher in the low-risk group, and the tumor purity (**Figure 6G**) being higher in the high-risk group. This observation aligns with the findings from the previous ssGSEA analysis. In general, the HRD low-risk group exhibited higher levels of immune cell infiltration, and this subgroup of patients was more suitable for immunotherapy.

### 3.7 Immunotherapy analysis and identification of potential small molecule compounds

**Figure 7:**
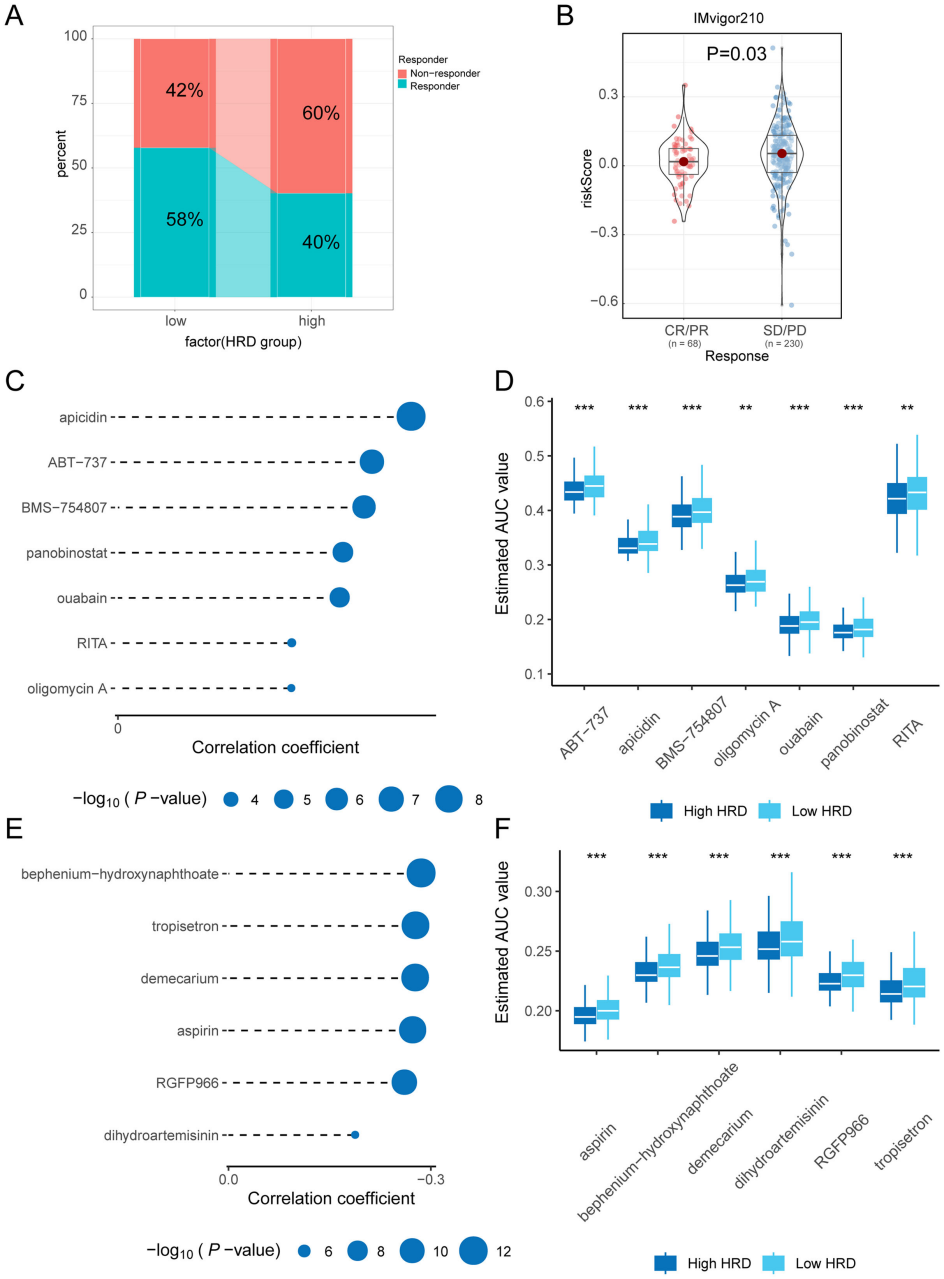
Immunotherapy and drug-sensitivity analysis. **(A)** Bar graph of percentage difference in immune response between high and low-risk groups of HRD predicted by TIDE; **(B)** Violin plot of the difference in efficacy based on the IMvigor210 bladder cancer Immunotherapy cohort; **(C)** Lollipop plot of correlation coefficients and p-values between seven small molecule compounds and HRD scores based on CTRP drug-susceptibility data; **(D)** Boxplot of the estimated AUC values of seven small molecule compounds between high and low-risk groups based on CTRP drug susceptibility data; **(E)** Lollipop plot of correlation coefficients and p-values between six small molecule compounds and HRD scores derived from PRISM drug-susceptibility data; **(F)** Boxplots of the estimated AUC values between the high and low-risk groups for the seven small molecule compounds based on PRISM drug susceptibility data. (HRD, Homologous recombination deficiency; TIDE, Tumor immune dysfunction and exclusion; CTRP, Cancer treatment response portal; AUC, Area Under the Curve; PRISM, Profiling relative inhibition simultaneously in mixtures).

To assess the difference in response to immunotherapy between the high and low-risk groups of HRD, we used the TIDE approach to predict the response to immunotherapy in subgroups that favored the low-risk group (**Figure 7A**). To confirm the reliability of our results, we performed differential analyses among patients with different immune responses in the IMvigor210 bladder cancer immunotherapy data set. Among them, CR/PR represents complete or partial response, and SD/PD represents stable or progressive disease. The risk score was significantly lower in the CR/PR group, this suggests that patients in the low-risk group may have a greater likelihood of responding positively to immunotherapy (**Figure 7B**). To find potential therapeutic agents that are effective in glioma patients, we used CTRP and PRISM combined with CMap comprehensive analysis to search for potential small-molecule drugs. We utilized data from CTRP and PRISM to identify drugs that could potentially be more effective for patients classified in the high-risk group, this resulted in seven CTRP-derived drugs (apicidin, ABT−737, BMS−754807, panobinostat, ouabain, RITA, and oligomycin A) and six prismatic drugs Derivatives (bephenium−hydroxynaphthoate, tropisetron, demecarium, aspirin, RGFP966, and dihydroartemisinin). The estimated AUC values for these drugs displayed a statistically significant negative correlation with the HRD score and were notably lower in the high-risk group (**Figures 7C–F**). Next, based on the differential expression profiles between two groups of glioma patients, the CMap tool was further applied to identify candidate compounds in the high-risk group of gliomas. By cross-referencing the findings from CTRP and PRISM, we identified six potential candidate compounds: apicidin, a histone deacetylase inhibitor; ABT−737, a Bcl-2 family inhibitor; BMS−754807, a tyrosine kinase inhibitor The inhibitor ouabain and the histone deacetylase (HDAC) inhibitors panobinostat and RITA (Reactivating p53 and Inducing Tumor Apoptosis). The corresponding targets of action and their mechanisms of action (MOA) are shown in **Table 1**. Panobinostat, achieving a CMap score of 84.92, exhibited pronounced sensitivity to glioma patients, highlighting its potential as a therapeutic option for those in the high-risk glioma group.

**Table 1:** Selected 6 therapeutic drugs, targets, and mechanisms of action in the Cmap database.

Score	ID	Name	Description	Target	MOA
84.92	BRD-K02130563	panobinostat	HDAC inhibitor	HDAC1, HDAC2, HDAC3, HDAC4, HDAC6, HDAC7, HDAC8, HDAC9	HDAC inhibitor
66.09	BRD-K13049116	BMS-754807	IGF-1 inhibitor	IGF1R, AKT1	IGF-1 inhibitor
58.51	BRD-A68930007	ouabain	ATPase inhibitor	ATP1A1, ATP1A2, ATP1A3, ATP1A4, ATP1B1, ATP1B2, ATP1B3, ATP1B4, FXYD2	ATPase inhibitor
52.27	BRD-K56301217	ABT-737	BCL inhibitor	BCL2, BCL2L1, BCL2L2	BCL inhibitor
66.32	BRD-K64606589	apicidin	HDAC inhibitor	HDAC1, HDAC10, HDAC11, HDAC2, HDAC3, HDAC4, HDAC5, HDAC6, HDAC7, HDAC8, HDAC9	HDAC inhibitor
78.87	BRD-K00317371	RITA	MDM inhibitor	MDM2, TXNRD1, TXNRD2	MDM inhibitor

### 3.8 Molecular docking

**Figure 8:**
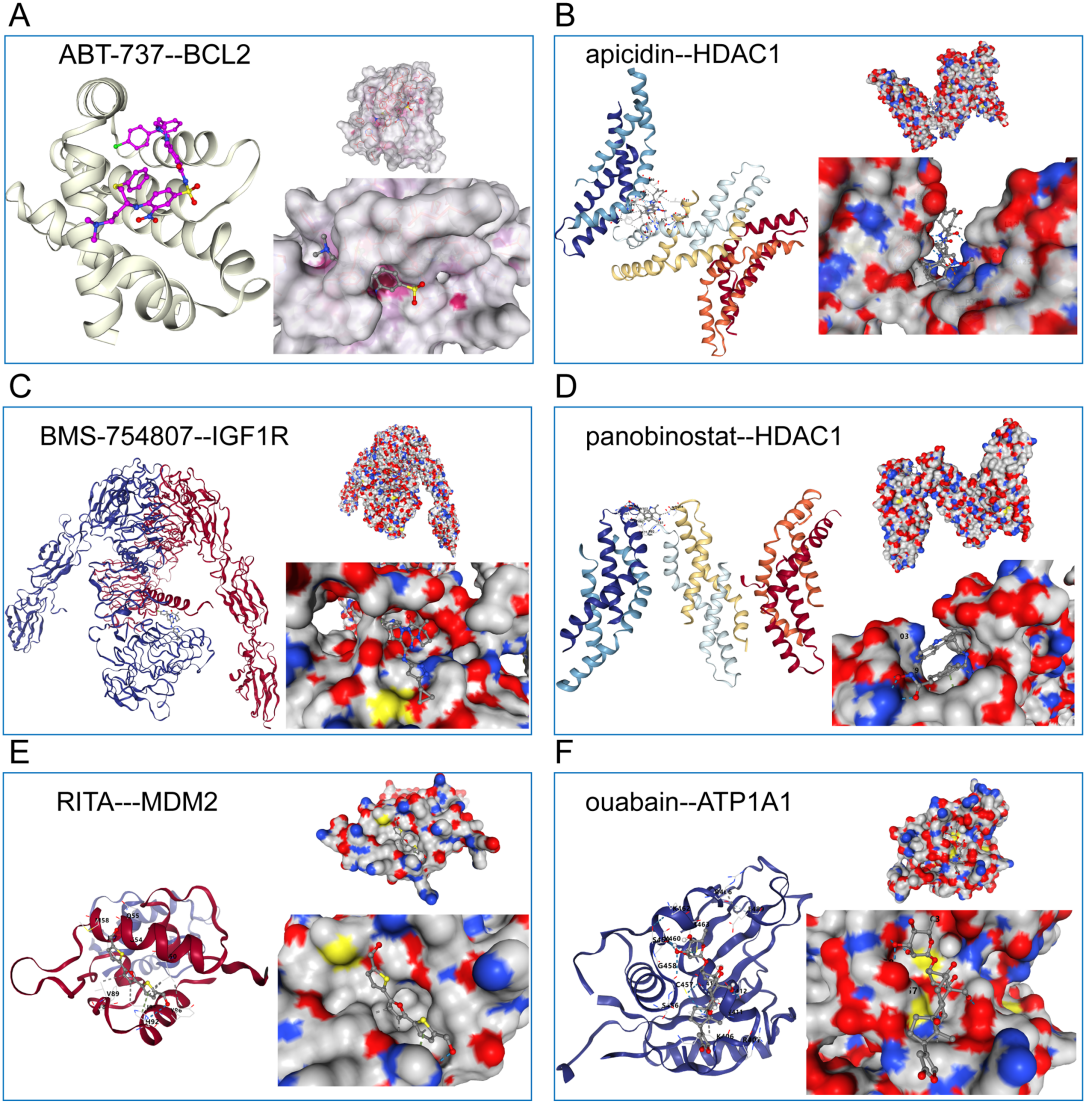
Schematic diagram of the molecular docking of six candidate drugs and their receptor interaction pairs. **(A)** Abt-737 interacts with BCL2 ligands; **(B)** Apicidin interacts with HDAC1 ligands; **(C)** BMS-754807 interacts with IGF1R ligands; **(D)** Phenobinostat interacts with HDAC1 ligands; **(E)** ITA interacts with MDM2 ligand-receptor; And **(F)** Ouabain interacts with ATP1A1 ligand-receptor. The left side represents the schematic diagram of the positions of the compound and the receptor protein skeleton, and the right side represents the molecular docking details of the ligand-receptor interaction pair.

We will get the above analysis of these six against each other with receptors based on CB - Dock2 (https://cadd.labshare.cn/cb-dock2/php/index.php) for the connection of potential drugs and receptor molecules with the target analysis and visualization. These include: ABT−737 interacting with BCL2 ligand receptor (**Figure 8A**), apicidin interacting with the HDAC1 ligand receptor (**Figure 8B**), BMS−754807 interacting with IGF1R ligand receptor (**Figure 8C**), panobinostat interacts with HDAC1 ligand receptor (**Figure 8D**), RITA interacts with MDM2 ligand-receptor Interaction pair (**Figure 8E**), ouabain and ATP1A1 ligand-receptor interaction pair (**Figure 8F**).

## 4 Discussion

The HRD score, deriving from the aggregation of LOH, TAI, and LST, has previously been recognized as a significant indicator in several types of cancer (35–37), notably, ovarian cancer patients with HRD have shown enhanced responses to platinum-based chemotherapy and PARPi (53). Additionally, the HRD score has demonstrated its potential in predicting the response of patients with triple-negative breast cancer to chemotherapy, and HRD-related signatures also hold potential for prognostic prediction and drug sensitivity assessment in gastric cancer patients (54, 55). In the context of glioma, the significance of HRD has not been extensively explored. Through a comprehensive data-driven analysis, we discerned that patients with glioma can be categorized into high and low-risk cohorts according to their HRD scores. Our research enabled the identification of potential HRD-associated signature genes and facilitated the development of a prognostic model to anticipate patient outcomes. We also investigated the immune infiltration, immunomodulators, and immune responses in glioma, offering an additional dimension to comprehend the implications of HRD in glioma. Furthermore, guided by our risk model, we identified drugs that might exhibit heightened efficacy against glioma and undertook molecular docking analyses, paving the way for innovative therapeutic strategies for glioma.

In this study, a significant association between elevated HRD scores and genomic instability was found. Such genomic instability is known to play a critical role in the progression of various cancers, contributing to tumor heterogeneity, resistance to therapy, and poor prognosis (56–59). The consistency of these markers with the HRD score further underscores the utility of the HRD score as a comprehensive metric for genomic instability. The prognostic significance of the HRD score in predicting patient outcomes was another noteworthy finding. By discerning an optimal cutoff value, the study effectively stratified glioma patients into HRD high and low-expression groups. The survival analysis, including OS, DSS, and PFI, suggested that patients with a higher HRD score faced a significantly worse prognosis. This is in line with previous studies in other malignancies where genomic instability was linked to aggressive tumor behavior and diminished survival rates (59, 60).

Based on the previous findings, we constructed an HRD-related prognostic model with the utilization of StepCox[both] and Lasso. The initial identification of 35 genes was narrowed down to a more streamlined set of 7 genes, including NKX6.3, PITX2, ZAR1, CD70, HOXA3, HOXB13, and HOXC9, which signifies the power of combining two algorithmic approaches. Among these 7 HRD-related genes, NKX6.3 was reported to be commonly observed with diminished or absent expression in gastric cancer, acts as a tumor-suppressor, inhibiting cell proliferation and promoting apoptosis. Studies indicate that the inhibition of PITX2 diminished the viability of liver cancer cells and decreased the capabilities of cell proliferation, migration, and invasion while enhancing cell-apoptosis (61). Moreover, PITX2 has been identified as a poor prognostic biomarker in breast cancer, colorectal cancer, and head and neck squamous cell carcinoma (62–64). Our study found for the first time that these two genes, as HRD-related genes, also play an important role in the prognosis and treatment of glioma. CD70 contributes to the recruitment and maintenance of the immunosuppressive microenvironment in GBM, concurrently facilitating pathways that promote tumor growth, it emerges as a promising immunotherapeutic target for recurrent GBM (65). This also hints at the possibility and rationality of our immune-related analysis based on the constructed risk model. When it comes to three homeobox genes, recent investigations have elucidated that some of them may act as prognostic biomarker associated with a poor outcome in glioma patients, and they could regulate cell proliferation, migration, autophagy and tumor progression of glioma (66–68). These studies provide evidence supporting the prognostic utility of our risk model. and our analysis also identifies promising areas for the advancement of future targeted therapeutics.

The paramount significance of our risk model rests in its superior predictive performance against pre-existing prognostic models for glioma in several datasets (42–46). With consistent results across diverse validation sets, its robustness stands stable. Current study further bridges the transitional gap, highlighting the risk model’s relevance not just in glioma but also in hepatocellular carcinoma, osteosarcoma, and epithelial ovarian cancer. This suggests a broader oncological relevance, potentially revolutionizing the management of various malignancies by providing a universally applicable prognostic tool.

Based on the constructed risk model, pathway enrichment and comparison of genomic signatures in groups rated as high risk and low risk were performed. It is noteworthy that the low-risk group’s enrichment in pathways like chemokine signaling and Toll-like receptor signaling suggests an immune response component, potentially indicating an immune-active tumor microenvironment (69–71). Such insights could offer therapeutic opportunities leveraging immunotherapy strategies. The evaluation of genetic variants underscores a profound level of genomic instability in the high-risk group. The elevated TMB, MSI sensor score, and overall genomic alteration scores in the high-risk patients align with the aggressive phenotype of their tumors. Genomic instability often equates with increased therapeutic vulnerabilities. However, it also poses challenges as tumors might rapidly adapt and develop resistance to treatments (57). The similarity in mutated driver genes across risk groups, yet with a higher percentage of gene mutations in the high-risk group, emphasizes this instability and suggests potential treatment targets for personalized therapies.

In the current study, we established that patients categorized as low-risk exhibited elevated levels of immune cell infiltration, suggesting that those classified as low-risk had a ‘hot’ and suppressed tumor immune microenvironment (TIME). The tumor microenvironment has emerged as a focal point of modern oncology, both as a prognostic marker and a therapeutic target. The findings of differential immune infiltration reinforce the evolving understanding of glioma’s complex relationship with host immunity. A ‘hot’ tumor, characterized by robust immune cell infiltration, has often been linked with better therapeutic outcomes, especially in the era of immunotherapy. Our findings, indicating pronounced immune cell presence in the low-risk group, not only provide a prognostic marker but also suggest potential therapeutic avenues, this is consistent with previous literature reports that pre-existing antitumor immunity is beneficial to tumor patient’s survival (72, 73). Conversely, high-risk group patients with “cold” TIME might be considered appropriate candidates for the administration of immunostimulatory agents like tumor vaccines, with the aim of enhancing the infiltration of tumor-targeting immune cells. As the patients in the high-risk group have higher HRD scores, PARP inhibitors could also be considered in combination therapy (74). The result of the upregulation of immunogenic cell death modulators in the low-risk group further fuels the hypothesis of these tumors being more amenable to immunotherapeutic strategies.

The observed variation in the effectiveness of immunotherapy across individuals classified as high and low risk highlights the importance of genetic factors in influencing the success of therapeutic interventions. The TIDE approach suggests a favorable response to immunotherapy in the low-risk group, corroborated further by the IMvigor210 bladder cancer dataset where the low-risk score was predominant among responders. This finding positions the HRD low-risk group as a potential beneficiary of immunotherapeutic interventions. Yet, the exploration of targeted small molecule compounds for the high-risk group was also performed. Leveraging a combination of CTRP, PRISM, and CMap comprehensive analyses, a suite of potential therapeutic agents was identified, each with distinct mechanisms of action. Notably, drugs like apicidin, a histone deacetylase inhibitor, and BMS−754807, a tyrosine kinase inhibitor, emerged as potential candidates, with panobinostat, another histone deacetylase inhibitor, being spotlighted due to its high sensitivity score. This compound functions as an epigenetic modulator by effectively the activity of histone deacetylase, hence leading to an increase in the acetylation of DNA-histone complexes. According to Van Veggel et al., this intervention obstructs many signals associated with tumor growth and progress, ultimately triggering cell apoptosis in specific cells (75). In addition, it has been shown in previous studies that increased histone acetylation renders cancer cells more susceptible to the impact of alkylating agents, such as TMZ (76).

The subsequent molecular docking analysis, utilizing CB-Dock2, provides a deeper understanding of the molecular interactions underpinning the efficacy of these compounds. For instance, ABT−737’s interaction with the BCL2 ligand-receptor reaffirms its established role as an apoptosis modulator in cancer therapy (77, 78). Given that BCL2 overexpression has been implicated in various cancer types as a driver of resistance, the drug’s efficacy in high-risk glioma patients underscores the importance of apoptosis modulation in this subgroup (79, 80). Similarly, BMS−754807’s interaction with the IGF1R ligand-receptor resonates with literature indicating the IGF signaling pathway’s involvement in tumorigenesis and resistance mechanisms. IGF1R inhibitors have recently gained traction in clinical trials for diverse malignancies, and their potential role in glioma, especially high-risk subsets, opens avenues for targeted therapeutic strategies (81–83). These findings offer mechanistic insights that could guide the optimization and application of these compounds in clinical settings.

There still exist several limitations within the scope of this study. Further preclinical investigations in glioma are needed to clarify the biological significance of genes in the risk model. The outcomes, including the predictive significance of the risk model and the identified possible small molecule drugs, are significantly dependent on the datasets employed. The potential impact of variations in data quality, processing methods, and inherent biases within datasets on the generalizability of the findings should be considered. Moreover, the small molecule compounds that have been found and their potential efficacy are derived from computer evaluations. Clinical trials play a crucial role in establishing the safety, effectiveness, and therapeutic capacity of interventions in individuals receiving medical treatment. In our following steps, efforts will be made to address these limitations through experimental investigation.

## 5 Conclusion

In this study, we delineated the significant prognostic capabilities of the HRD score in stratifying glioma patients into distinct risk categories. By diving deeper into the genetic intricacies of these risk-based groups, a set of signature genes was identified, enabling the construction of a robust prognostic model tailored for glioma. This innovative approach offers a leap forward in individualized patient management, paving the way for more precise therapeutic interventions. Furthermore, our findings shed light on the pronounced differences in immune infiltration and regulation between the risk groups, offering valuable insights into potential immunotherapeutic strategies. The drug screening and molecular docking analyses also unveiled promising therapeutic agents, suggesting novel avenues for glioma treatment. Overall, our research has furnished both theoretical foundations and practical avenues for understanding and combating glioma more effectively.

## Data Availability

The original contributions presented in the study are included in the article/**Supplementary Material**. Further inquiries can be directed to the corresponding authors.

## References

[1] Ostrom QT Price M Neff C Cioffi G Waite KA Kruchko C. CBTRUS statistical report: primary brain and other central nervous system tumors diagnosed in the United States in 2015-2019. Neuro Oncol. (2022) 24:V1–V95. doi: 10.1093/NEUONC/NOAC202 36196752 PMC9533228

[2] Omuro A DeAngelis LM. Glioblastoma and other Malignant gliomas: a clinical review. JAMA. (2013) 310:1842–50. doi: 10.1001/JAMA.2013.280319 24193082

[3] Ohgaki H Kleihues P. Population-based studies on incidence, survival rates, and genetic alterations in astrocytic and oligodendroglial gliomas. J Neuropathol Exp Neurol. (2005) 64:479–89. doi: 10.1093/JNEN/64.6.479 15977639

[4] Tan AC Ashley DM López GY Malinzak M Friedman HS Khasraw M. Management of glioblastoma: State of the art and future directions. CA Cancer J Clin. (2020) 70:299–312. doi: 10.3322/CAAC.21613 32478924

[5] Louis DN Perry A Wesseling P Brat DJ Cree IA Figarella-Branger D. The 2021 WHO classification of tumors of the central nervous system: a summary. Neuro Oncol. (2021) 23:1231. doi: 10.1093/NEUONC/NOAB106 34185076 PMC8328013

[6] Kan LK Drummond K Hunn M Williams D O’Brien TJ Monif M. Potential biomarkers and challenges in glioma diagnosis, therapy and prognosis. BMJ Neurol Open. (2020) 2:69. doi: 10.1136/BMJNO-2020-000069 PMC7871709 33681797

[7] Scully R Panday A Elango R Willis NA. DNA double-strand break repair-pathway choice in somatic mammalian cells. Nat Rev Mol Cell Biol 2019 20:11. (2019) 20:698–714. doi: 10.1038/s41580-019-0152-0 PMC7315405 31263220

[8] Royfman R Whiteley E Noe O Morand S Creeden J Stanbery L. BRCA1/2 signaling and homologous recombination deficiency in breast and ovarian cancer. Future Oncol. (2021) 17:2817–30. doi: 10.2217/FON-2021-0072 34058833

[9] Blanc-Durand F Tang R Pommier M Nashvi M Cotteret S Genestie C. Clinical relevance of BRCA1 promoter methylation testing in patients with ovarian cancer. Clin Cancer Res. (2023) 29:3124–9. doi: 10.1158/1078-0432.CCR-22-3328 37067532

[10] Leibowitz BD Dougherty BV Bell JSK Kapilivsky J Michuda J Sedgewick AJ. Validation of genomic and transcriptomic models of homologous recombination deficiency in a real-world pan-cancer cohort. BMC Cancer. (2022) 22:1–17. doi: 10.1186/S12885-022-09669-Z PMC9148513 35643464

[11] Yamamoto H Hirasawa A. Homologous recombination deficiencies and hereditary tumors. Int J Mol Sci. (2021) 23:348. doi: 10.3390/IJMS23010348 35008774 PMC8745585

[12] Jeggo PA Pearl LH Carr AM. DNA repair, genome stability and cancer: a historical perspective. Nat Rev Cancer. (2016) 16:35–42. doi: 10.1038/NRC.2015.4 26667849

[13] Yang D Khan S Sun Y Hess K Shmulevich I Sood AK. Association of BRCA1 and BRCA2 mutations with survival, chemotherapy sensitivity, and gene mutator phenotype in patients with ovarian cancer. JAMA. (2011) 306:1557–65. doi: 10.1001/JAMA.2011.1456 PMC4159096 21990299

[14] Tumiati M Hietanen S Hynninen J Pietila E Farkkil A Kaipio K. A functional homologous recombination assay predicts primary chemotherapy response and long-term survival in ovarian cancer patients. Clin Cancer Res. (2018) 24:4482–93. doi: 10.1158/1078-0432.CCR-17-3770 29858219

[15] Lord CJ Ashworth A. PARP inhibitors: Synthetic lethality in the clinic. Science. (2017) 355:1152–8. doi: 10.1126/SCIENCE.AAM7344 PMC6175050 28302823

[16] Robson ME Tung N Conte P Im SA Senkus E Xu B. OlympiAD final overall survival and tolerability results: Olaparib versus chemotherapy treatment of physician’s choice in patients with a germline BRCA mutation and HER2-negative metastatic breast cancer. Ann Oncol. (2019) 30:558–66. doi: 10.1093/ANNONC/MDZ012 PMC6503629 30689707

[17] Ohba S Yamashiro K Hirose Y. Inhibition of DNA repair in combination with temozolomide or dianhydrogalactiol overcomes temozolomide-resistant glioma cells. Cancers (Basel). (2021) 13:2570. doi: 10.3390/CANCERS13112570 34073837 PMC8197190

[18] Lotan TL Kaur HB Salles DC Murali S Schaeffer EM Lanchbury JS. Homologous recombination deficiency (HRD) score in germline BRCA2- versus ATM-altered prostate cancer. Mod Pathol. (2021) 34:1185–93. doi: 10.1038/S41379-020-00731-4 PMC8154637 33462368

[19] Loibl S Weber KE Timms KM Elkin EP Hahnen E Fasching PA. Survival analysis of carboplatin added to an anthracycline/taxane-based neoadjuvant chemotherapy and HRD score as predictor of response-final results from GeparSixto. Ann Oncol. (2018) 29:2341–7. doi: 10.1093/ANNONC/MDY460 30335131

[20] Cadoo K Simpkins F Mathews C Liu YL Provencher D McCormick C. Olaparib treatment for platinum-sensitive relapsed ovarian cancer by BRCA mutation and homologous recombination deficiency status: Phase II LIGHT study primary analysis. Gynecol Oncol. (2022) 166:425–31. doi: 10.1016/J.YGYNO.2022.06.017 PMC9909678 35803835

[21] Luo J Pan M Mo K Mao Y Zou D. Emerging role of artificial intelligence in diagnosis, classification and clinical management of glioma. Semin Cancer Biol. (2023) 91:110–23. doi: 10.1016/J.SEMCANCER.2023.03.006 36907387

[22] Wang X Huang Y Li S Zhang H. Integrated machine learning methods identify FNDC3B as a potential prognostic biomarker and correlated with immune infiltrates in glioma. Front Immunol. (2022) 13:1027154. doi: 10.3389/FIMMU.2022.1027154 36275754 PMC9582524

[23] Zhang N Dai Z Wu W Wang Z Cao H Zhang Y. The predictive value of monocytes in immune microenvironment and prognosis of glioma patients based on machine learning. Front Immunol. (2021) 12:656541. doi: 10.3389/FIMMU.2021.656541 33959130 PMC8095378

[24] Della Pepa GM Caccavella VM Menna G Ius T Auricchio AM Sabatino G. Machine learning-based prediction of early recurrence in glioblastoma patients: A glance towards precision medicine. Neurosurgery. (2021) 89:873–83. doi: 10.1093/NEUROS/NYAB320 34459917

[25] Zhang H Zhang N Wu W Zhou R Li S Wang Z. Machine learning-based tumor-infiltrating immune cell-associated lncRNAs for predicting prognosis and immunotherapy response in patients with glioblastoma. Brief Bioinform. (2022) 23:1–14. doi: 10.1093/BIB/BBAC386 36136350

[26] Goldman MJ Craft B Hastie M Repečka K McDade F Kamath A. Visualizing and interpreting cancer genomics data via the Xena platform. Nat Biotechnol. (2020) 38:675–8. doi: 10.1038/S41587-020-0546-8 PMC7386072 32444850

[27] Thorsson V Gibbs DL Brown SD Wolf D Bortone DS Ou Yang TH. The immune landscape of cancer. Immunity. (2018) 48:812–830.e14. doi: 10.1016/J.IMMUNI.2018.03.023 29628290 PMC5982584

[28] Mayakonda A Lin DC Assenov Y Plass C Koeffler HP. Maftools: efficient and comprehensive analysis of somatic variants in cancer. Genome Res. (2018) 28:1747–56. doi: 10.1101/GR.239244.118 PMC6211645 30341162

[29] Barrett T Wilhite SE Ledoux P Evangelista C Kim IF Tomashevsky M. NCBI GEO: archive for functional genomics data sets–update. Nucleic Acids Res. (2013) 41:D991–5. doi: 10.1093/NAR/GKS1193 PMC3531084 23193258

[30] Gusev Y Bhuvaneshwar K Song L Zenklusen JC Fine H Madhavan S. The REMBRANDT study, a large collection of genomic data from brain cancer patients. Sci Data. (2018) 5:1–9. doi: 10.1038/SDATA.2018.158 PMC6091243 30106394

[31] Erdem-Eraslan L Van Den Bent MJ Hoogstrate Y Naz-Khan H Stubbs A van der Spek P. Identification of patients with recurrent glioblastoma who may benefit from combined bevacizumab and CCNU therapy: A report from the BELOB trial. Cancer Res. (2016) 76:525–34. doi: 10.1158/0008-5472.CAN-15-0776 26762204

[32] Zhao Z Zhang KN Wang Q Li G Zeng F Zhang Y. Chinese glioma genome atlas (CGGA): A comprehensive resource with functional genomic data from chinese glioma patients. Genomics Proteomics Bioinf. (2021) 19:1–12. doi: 10.1016/J.GPB.2020.10.005 PMC8498921 33662628

[33] Zhang K Liu X Li G Chang X Li S Chen J. Clinical management and survival outcomes of patients with different molecular subtypes of diffuse gliomas in China (2011-2017): a multicenter retrospective study from CGGA. Cancer Biol Med. (2022) 19:1460–76. doi: 10.20892/J.ISSN.2095-3941.2022.0469 PMC9630520 36350010

[34] Zhao Z Meng F Wang W Wang Z Zhang C Jiang T. Comprehensive RNA-seq transcriptomic profiling in the Malignant progression of gliomas. Sci Data. (2017) 4:1–7. doi: 10.1038/SDATA.2017.24 PMC5349247 28291232

[35] Abkevich V Timms KM Hennessy BT Potter J Carey MS Meyer LA. Patterns of genomic loss of heterozygosity predict homologous recombination repair defects in epithelial ovarian cancer. Br J Cancer. (2012) 107:1776–82. doi: 10.1038/BJC.2012.451 PMC3493866 23047548

[36] Birkbak NJ Wang ZC Kim JY Eklund AC Li Q Tian R. Telomeric allelic imbalance indicates defective DNA repair and sensitivity to DNA-damaging agents. Cancer Discov. (2012) 2:366–75. doi: 10.1158/2159-8290.CD-11-0206 PMC3806629 22576213

[37] Popova T Manié E Rieunier G Caux-Moncoutier V Tirapo C Dubois T. Ploidy and large-scale genomic instability consistently identify basal-like breast carcinomas with BRCA1/2 inactivation. Cancer Res. (2012) 72:5454–62. doi: 10.1158/0008-5472.CAN-12-1470 22933060

[38] Ritchie ME Phipson B Wu D Hu Y Law CW Shi W. limma powers differential expression analyses for RNA-sequencing and microarray studies. Nucleic Acids Res. (2015) 43:e47. doi: 10.1093/NAR/GKV007 25605792 PMC4402510

[39] Wang Y Ma X Xu E Huang Z Yang C Zhu K. Identifying squalene epoxidase as a metabolic vulnerability in high-risk osteosarcoma using an artificial intelligence-derived prognostic index. Clin Transl Med. (2024) 14:e1586. doi: 10.1002/CTM2.1586 38372422 PMC10875711

[40] Chen Y Wang B Zhao Y Shao X Wang M Ma F. Metabolomic machine learning predictor for diagnosis and prognosis of gastric cancer. Nat Commun. (2024) 15:1–13. doi: 10.1038/S41467-024-46043-Y PMC10891053 38395893

[41] Clift AK Dodwell D Lord S Petrou S Brady M Collins GS. Development and internal-external validation of statistical and machine learning models for breast cancer prognostication: cohort study. BMJ. (2023) 381:e073800. doi: 10.1136/BMJ-2022-073800 37164379 PMC10170264

[42] Tong S Xia M Xu Y Sun Q Ye L Cai J. Identification and validation of a 17-gene signature to improve the survival prediction of gliomas. Front Immunol. (2022) 13:1000396. doi: 10.3389/FIMMU.2022.1000396 36248799 PMC9556650

[43] Cai J Hu Y Ye Z Ye L Gao L Wang Y. Immunogenic cell death-related risk signature predicts prognosis and characterizes the tumour microenvironment in lower-grade glioma. Front Immunol. (2022) 13:1011757. doi: 10.3389/FIMMU.2022.1011757 36325335 PMC9618960

[44] Tan YQ Li YT Yan TF Xu Y Liu BH Yang JA. Six immune associated genes construct prognostic model evaluate low-grade glioma. Front Immunol. (2020) 11:606164. doi: 10.3389/FIMMU.2020.606164 33408717 PMC7779629

[45] Zhang H Huang Y Yang E Gao X Zou P Sun J. Identification of a fibroblast-related prognostic model in glioma based on bioinformatics methods. Biomolecules. (2022) 12:1598. doi: 10.3390/BIOM12111598 36358948 PMC9687522

[46] Li X Wang Y Wu W Xiang J Wang M Yu H. A novel DNA damage and repair-related gene signature to improve predictive capacity of overall survival for patients with gliomas. J Cell Mol Med. (2022) 26:3736–50. doi: 10.1111/JCMM.17406 PMC9258707 35615996

[47] Yu G Wang LG Han Y He QY. ClusterProfiler: An R package for comparing biological themes among gene clusters. OMICS. (2012) 16:284–7. doi: 10.1089/omi.2011.0118 PMC3339379 22455463

[48] Newman AM Steen CB Liu CL Gentles AJ Chaudhuri AA Scherer F. Determining cell type abundance and expression from bulk tissues with digital cytometry. Nat Biotechnol. (2019) 37:773–82. doi: 10.1038/S41587-019-0114-2 PMC6610714 31061481

[49] Jiang P Gu S Pan D Fu J Sahu A Hu X. Signatures of T cell dysfunction and exclusion predict cancer immunotherapy response. Nat Med. (2018) 24:1550–8. doi: 10.1038/S41591-018-0136-1 PMC6487502 30127393

[50] Yang C Huang X Li Y Chen J Lv Y Dai S. Prognosis and personalized treatment prediction in TP53-mutant hepatocellular carcinoma: an in silico strategy towards precision oncology. Brief Bioinform. (2021) 22:1–13. doi: 10.1093/BIB/BBAA164 32789496

[51] Subramanian A Narayan R Corsello SM Peck DD Natoli TE Lu X. A next generation connectivity map: L1000 platform and the first 1,000,000 profiles. Cell. (2017) 171:1437–1452.e17. doi: 10.1016/J.CELL.2017.10.049 29195078 PMC5990023

[52] Malta TM Sokolov A Gentles AJ Burzykowski T Poisson L Weinstein JN. Machine learning identifies stemness features associated with oncogenic dedifferentiation. Cell. (2018) 173:338–354.e15. doi: 10.1016/J.CELL.2018.03.034 29625051 PMC5902191

[53] da Cunha Colombo Bonadio RR Fogace RN Miranda VC Diz MDPE. Homologous recombination deficiency in ovarian cancer: a review of its epidemiology and management. Clinics (Sao Paulo). (2018) 73:e450s. doi: 10.6061/CLINICS/2018/E450S 30133561 PMC6096977

[54] Wu X Wang Q Liu P Sun L Wang Y. Potential value of the homologous recombination deficiency signature we developed in the prognosis and drug sensitivity of gastric cancer. Front Genet. (2022) 13:1026871/FULL. doi: 10.3389/FGENE.2022.1026871/FULL 36468004 PMC9709314

[55] Melinda LT Kirsten MT Julia R Bryan H Gordon BM Kristin CJ. Homologous recombination deficiency (HRD) score predicts response to platinum-containing neoadjuvant chemotherapy in patients with triple-negative breast cancer. Clin Cancer Res. (2016) 22:3764–73. doi: 10.1158/1078-0432.CCR-15-2477 PMC6773427 26957554

[56] Yang H Zhang W Ding J Hu J Sun Y Peng W. A novel genomic instability-derived lncRNA signature to predict prognosis and immune characteristics of pancreatic ductal adenocarcinoma. Front Immunol. (2022) 13:970588. doi: 10.3389/FIMMU.2022.970588 36148233 PMC9486402

[57] Dharanipragada P Zhang X Liu S Lomeli SH Hong A Wang Y. Blocking genomic instability prevents acquired resistance to MAPK inhibitor therapy in melanoma. Cancer Discov. (2023) 13:880–909. doi: 10.1158/2159-8290.CD-22-0787 36700848 PMC10068459

[58] Raynaud F Mina M Tavernari D Ciriello G. Pan-cancer inference of intra-tumor heterogeneity reveals associations with different forms of genomic instability. PLoS Genet. (2018) 14:e1007669. doi: 10.1371/JOURNAL.PGEN.1007669 30212491 PMC6155543

[59] Rumenapp C Smida J Gonzalez-Vasconcellos I Baumhoer D Malfoy B Hadj-Hamou N-S. Secondary radiation-induced bone tumours demonstrate a high degree of genomic instability predictive of a poor prognosis. Curr Genomics. (2012) 13:433–7. doi: 10.2174/138920212802510420 PMC3426777 23450216

[60] Malihi PD Graf RP Rodriguez A Ramesh N Lee J Sutton R. Single-cell circulating tumor cell analysis reveals genomic instability as a distinctive feature of aggressive prostate cancer. Clin Cancer Res. (2020) 26:4143–53. doi: 10.1158/1078-0432.CCR-19-4100 PMC8043601 32341031

[61] Tuerxun K Zhang S Zhang Y. Downregulation of PITX2 inhibits the proliferation and migration of liver cancer cells and induces cell apoptosis. Open Life Sci. (2021) 16:1322. doi: 10.1515/BIOL-2021-0133 35071766 PMC8724353

[62] Sailer V Gevensleben H Dietrich J Goltz D Kristiansen G Bootz F. Clinical performance validation of PITX2 DNA methylation as prognostic biomarker in patients with head and neck squamous cell carcinoma. PLoS One. (2017) 12:e0179412. doi: 10.1371/JOURNAL.PONE.0179412 28617833 PMC5472307

[63] Semaan A Uhl B Branchi V Lingohr P Bootz F Kristiansen G. Significance of PITX2 promoter methylation in colorectal carcinoma prognosis. Clin Colorectal Cancer. (2018) 17:e385–93. doi: 10.1016/J.CLCC.2018.02.008 29580650

[64] Nimmrich I Sieuwerts AM Meijer-Van Gelder ME Schwope I Bolt-De Vries J Harbeck N. DNA hypermethylation of PITX2 is a marker of poor prognosis in untreated lymph node-negative hormone receptor-positive breast cancer patients. Breast Cancer Res Treat. (2008) 111:429–37. doi: 10.1007/S10549-007-9800-8 17965955

[65] Seyfrid M Maich WT Shaikh VM Tatari N Upreti D Piyasena D. Original research: CD70 as an actionable immunotherapeutic target in recurrent glioblastoma and its microenvironment. J Immunother Cancer. (2022) 10:3289. doi: 10.1136/JITC-2021-003289 PMC8753449 35017149

[66] Yang R Zhang G Dong Z Wang S Li Y Lian F. Homeobox A3 and KDM6A cooperate in transcriptional control of aerobic glycolysis and glioblastoma progression. Neuro Oncol. (2023) 25:635–47. doi: 10.1093/NEUONC/NOAC231 PMC10076951 36215227

[67] Xuan F Huang M Liu W Ding H Yang L Cui H. Homeobox C9 suppresses Beclin1-mediated autophagy in glioblastoma by directly inhibiting the transcription of death-associated protein kinase 1. Neuro Oncol. (2016) 18:819. doi: 10.1093/NEUONC/NOV281 26582930 PMC4864258

[68] Wang X Sun Y Xu T Qian K Huang B Zhang K. HOXB13 promotes proliferation, migration, and invasion of glioblastoma through transcriptional upregulation of lncRNA HOXC-AS3. J Cell Biochem. (2019) 120:15527–37. doi: 10.1002/JCB.28819 31062400

[69] Deng S Zhu S Qiao Y Liu YJ Chen W Zhao G. Recent advances in the role of toll-like receptors and TLR agonists in immunotherapy for human glioma. Protein Cell. (2014) 5:899–911. doi: 10.1007/S13238-014-0112-6 25411122 PMC4259890

[70] Huang Y Zhang Q Lubas M Yuan Y Yalcin F Efe IE. Synergistic toll-like receptor 3/9 signaling affects properties and impairs glioma-promoting activity of microglia. J Neurosci. (2020) 40:6428–43. doi: 10.1523/JNEUROSCI.0666-20.2020 PMC7424875 32631940

[71] Dastmalchi F Karachi A Yang C Azari H Sayour EJ Dechkovskaia A. Sarcosine promotes trafficking of dendritic cells and improves efficacy of anti-tumor dendritic cell vaccines via CXC chemokine family signaling. J Immunother Cancer. (2019) 7:321. doi: 10.1186/S40425-019-0809-4 31753028 PMC6873439

[72] Li B Cui Y Nambiar DK Sunwoo JB Li R. The immune subtypes and landscape of squamous cell carcinoma. Clin Cancer Res. (2019) 25:3528–37. doi: 10.1158/1078-0432.CCR-18-4085 PMC6571041 30833271

[73] Rooney MS Shukla SA Wu CJ Getz G Hacohen N. Molecular and genetic properties of tumors associated with local immune cytolytic activity. Cell. (2015) 160:48–61. doi: 10.1016/J.CELL.2014.12.033 25594174 PMC4856474

[74] Galland L Roussot N Desmoulins I Mayeur D Kaderbhai C Ilie S. Clinical utility of genomic tests evaluating homologous recombination repair deficiency (HRD) for treatment decisions in early and metastatic breast cancer. Cancers (Basel). (2023) 15:1299. doi: 10.3390/CANCERS15041299 36831640 PMC9954086

[75] Van Veggel M Westerman E Hamberg P. Clinical pharmacokinetics and pharmacodynamics of panobinostat. Clin Pharmacokinet. (2018) 57:21–9. doi: 10.1007/S40262-017-0565-X 28667459

[76] Stiborova M Eckschlager T Poljakova J Hrabeta J Adam V Kizek R. The synergistic effects of DNA-targeted chemotherapeutics and histone deacetylase inhibitors as therapeutic strategies for cancer treatment. Curr Med Chem. (2012) 19:4218–38. doi: 10.2174/092986712802884286 22680633

[77] Dong D Dong Y Fu J Lu S Yuan C Xia M. Bcl2 inhibitor ABT737 reverses the Warburg effect via the Sirt3-HIF1α axis to promote oxidative stress-induced apoptosis in ovarian cancer cells. Life Sci. (2020) 255:117846. doi: 10.1016/J.LFS.2020.117846 32470451

[78] Choi JE Woo SM Min KJ Kang SH Lee SJ Kwon TK. Combined treatment with ABT-737 and VX-680 induces apoptosis in Bcl-2- and c-FLIP-overexpressing breast carcinoma cells. Oncol Rep. (2015) 33:1395–401. doi: 10.3892/OR.2015.3728 25592064

[79] Watanabe A Yasuhira S Inoue T Kasai S Shibazaki M Takahashi K. BCL2 and BCLxL are key determinants of resistance to antitubulin chemotherapeutics in melanoma cells. Exp Dermatol. (2013) 22:518–23. doi: 10.1111/EXD.12185 23802633

[80] Cheong HT Xu F Choy CT Hui CWC Mok TSK Wong CH. Upregulation of Bcl2 in NSCLC with acquired resistance to EGFR-TKI. Oncol Lett. (2018) 15:901–7. doi: 10.3892/OL.2017.7377 PMC5772989 29422965

[81] Simpson AD Soo YWJ Rieunier G Aleksic T Ansorge O Jones C. Type 1 IGF receptor associates with adverse outcome and cellular radioresistance in paediatric high-grade glioma. Br J Cancer. (2020) 122:624–9. doi: 10.1038/S41416-019-0677-1 PMC7054265 31857716

[82] De Billy E Pellegrino M Orlando D Pericoli G Ferretti R Businaro P. Dual IGF1R/IR inhibitors in combination with GD2-CAR T-cells display a potent anti-tumor activity in diffuse midline glioma H3K27M-mutant. Neuro Oncol. (2022) 24:1150–63. doi: 10.1093/NEUONC/NOAB300 PMC9248389 34964902

[83] Xue L Chen F Yue F Camacho L Kothapalli S Wei G. Metformin and an insulin/IGF-1 receptor inhibitor are synergistic in blocking growth of triple-negative breast cancer. Breast Cancer Res Treat. (2021) 185:73–84. doi: 10.1007/S10549-020-05927-5 32940848 PMC7855212

