# Supplementary material for: Development of a prognostic model related to homologous recombination deficiency in glioma based on multiple machine learning
Source: Front Immunol. 2024 Oct 7;15:1452097. doi: 10.3389/fimmu.2024.1452097 (PMC11491349; doi:10.3389/fimmu.2024.1452097)
Supplement: Supplementary file 1 [file DataSheet1.docx]

# Supplementary Figures and Tables

# Supplementary Figures

**
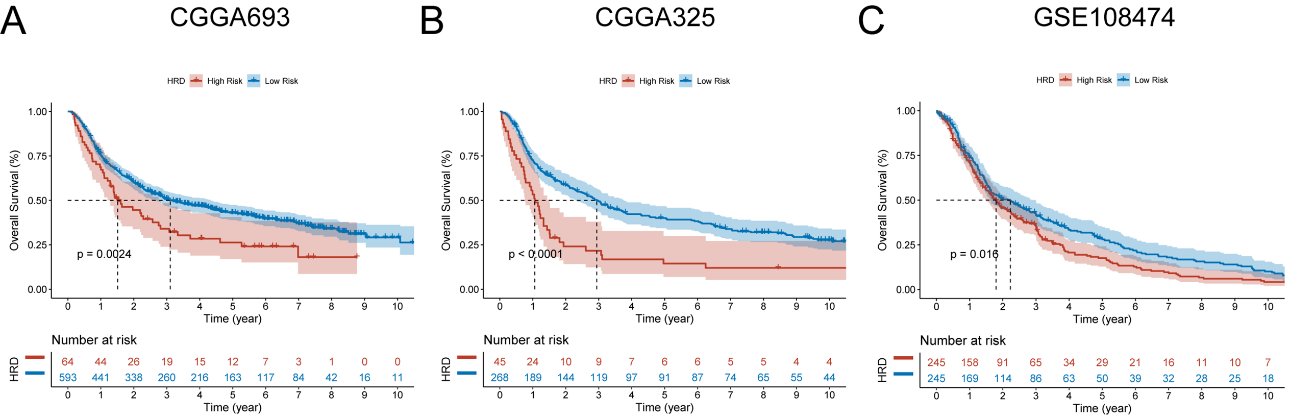
**

**Supplementary Figure 1. Survival Differences between high-risk and low-risk groups for HRD**

(a) KM curve of patients in the high and low-risk group of CGGA-693 in the validation set. (b) KM curve of patients in the high and low-risk group of the validation set CGGA-325. (c) KM curve of patients in the high and low-risk group of the validation set GSE108474.


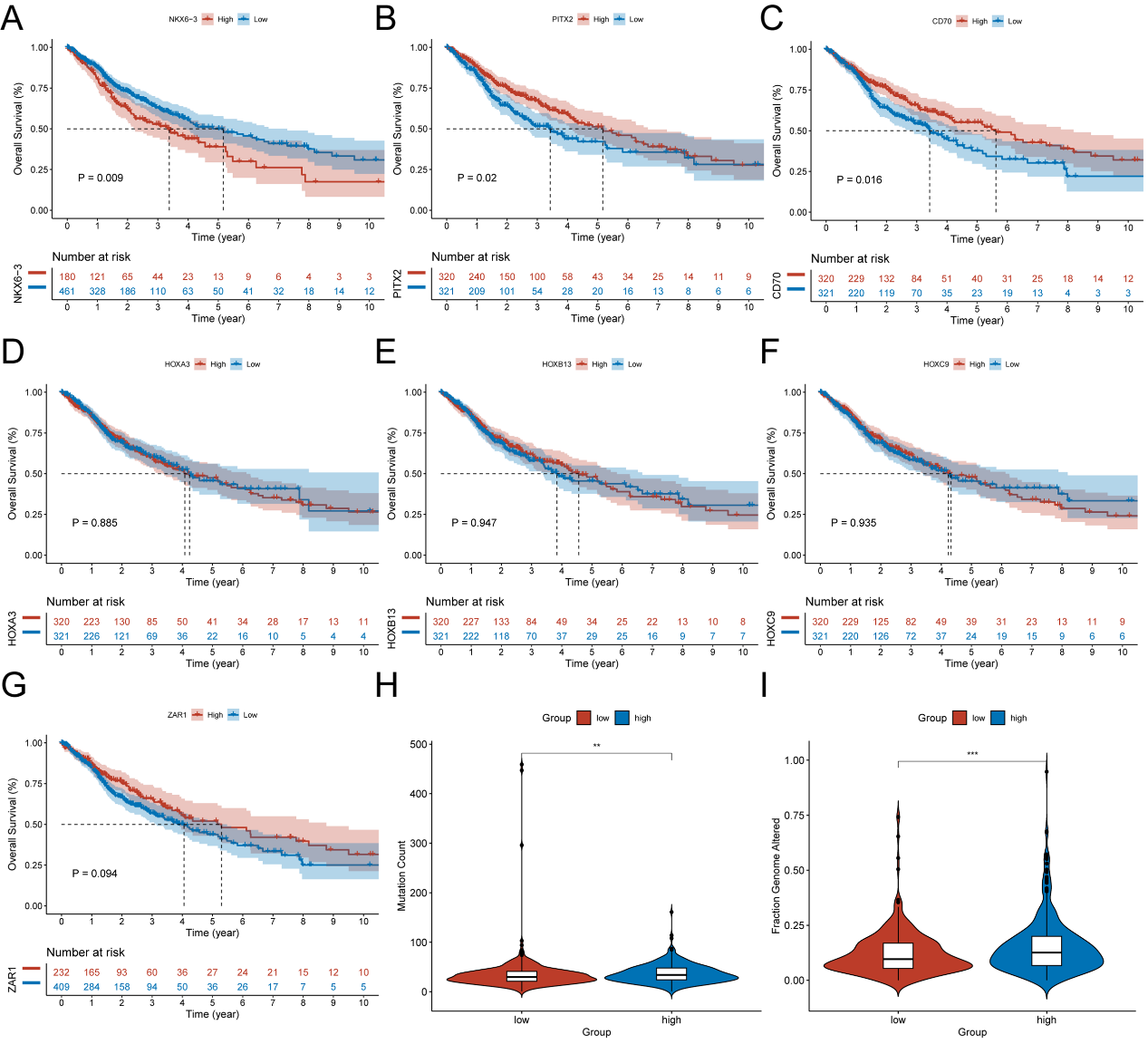


**Supplementary Figure 2. Survival curves related to the expression of seven key genes and genomic differences between high and low HRD expression groups.**

(a) NKX6.3 gene expression-related survival curves. (b) PITX2 gene expression-related survival curve. (c) CD70 gene expression-related survival curve. (d) Survival curves related to gene expression of OXA3. (e) Survival curve of HOXB13 gene expression. (f) Survival curve of HOXC9 gene expression. (g) ZAR1 gene expression-related survival curve. (h) Violin plot of mutation counts the difference between HRD high and low expression groups. (i) Violin plot of the difference in genomic alteration scores between HRD high and low expression groups.

# Supplementary Tables

Supplementary Table S1: Details of datasets used.

| Dataset | Number of Samples | Tumor Type | Data Type |
| --- | --- | --- | --- |
| CGGA_693 | 657 | Gliomas | Expression profiling by array |
| CGGA_325 | 313 | Gliomas | Expression profiling by array |
| GSE108474 | 550 | Gliomas | Expression profiling by array |
| GSE72951 | 112 | Gliomas | Expression profiling by array |
| IMvigor210 | 41 | Bladder Cancer | Expression profiling by array |
| TCGA-GBMLGG | 641 | Gliomas | RNA-seq |
| TCGA-LIHC | 363 | Liver Hepatocellular Carcinoma | RNA-seq |
| TARGET-OS | 85 | Osteosarcoma | RNA-seq |
| TCGA-OV | 353 | Ovarian Cancer | RNA-seq |

Supplementary Table S2: Homologous recombination deficiency score in TCGA-GBMLGG patients.

| ID | AI1 | LST1 | LOH | HRD | Cancer type |
| --- | --- | --- | --- | --- | --- |
| TCGA-02-2483-01A | 9 | 6 | 7 | 22 | GBMLGG |
| TCGA-02-2485-01A | 1 | 2 | 0 | 3 | GBMLGG |
| TCGA-02-2486-01A | 1 | 5 | 3 | 9 | GBMLGG |
| TCGA-06-1804-01A | 0 | 3 | 1 | 4 | GBMLGG |
| TCGA-06-2557-01A | 3 | 4 | 2 | 9 | GBMLGG |
| TCGA-06-2558-01A | 4 | 6 | 5 | 15 | GBMLGG |
| TCGA-06-2559-01A | 2 | 2 | 2 | 6 | GBMLGG |
| TCGA-06-2561-01A | 3 | 1 | 2 | 6 | GBMLGG |
| TCGA-06-2562-01A | 3 | 4 | 3 | 10 | GBMLGG |
| TCGA-06-2563-01A | 4 | 6 | 3 | 13 | GBMLGG |
| TCGA-06-2564-01A | 0 | 0 | 0 | 0 | GBMLGG |
| TCGA-06-2565-01A | 3 | 4 | 4 | 11 | GBMLGG |
| TCGA-06-2567-01A | 1 | 2 | 2 | 5 | GBMLGG |
| TCGA-06-2569-01A | 17 | 7 | 7 | 31 | GBMLGG |
| TCGA-06-2570-01A | 3 | 3 | 3 | 9 | GBMLGG |
| TCGA-06-5408-01A | 4 | 3 | 3 | 10 | GBMLGG |
| TCGA-06-5411-01A | 0 | 1 | 2 | 3 | GBMLGG |
| TCGA-06-5412-01A | 0 | 0 | 0 | 0 | GBMLGG |
| TCGA-06-5413-01A | 3 | 3 | 2 | 8 | GBMLGG |
| TCGA-06-5414-01A | 1 | 1 | 1 | 3 | GBMLGG |
| TCGA-06-5415-01A | 3 | 6 | 4 | 13 | GBMLGG |
| TCGA-06-5416-01A | 3 | 4 | 1 | 8 | GBMLGG |
| TCGA-06-5418-01A | 2 | 3 | 3 | 8 | GBMLGG |
| TCGA-06-5856-01A | 2 | 2 | 8 | 12 | GBMLGG |
| TCGA-06-5858-01A | 1 | 0 | 0 | 1 | GBMLGG |
| TCGA-06-5859-01A | 15 | 11 | 11 | 37 | GBMLGG |
| TCGA-12-3650-01A | 0 | 0 | 0 | 0 | GBMLGG |
| TCGA-12-3652-01A | 2 | 3 | 2 | 7 | GBMLGG |
| TCGA-12-3653-01A | 1 | 6 | 5 | 12 | GBMLGG |
| TCGA-12-5295-01A | 8 | 4 | 2 | 14 | GBMLGG |
| TCGA-12-5299-01A | 2 | 0 | 3 | 5 | GBMLGG |
| TCGA-14-1034-01A | 4 | 2 | 2 | 8 | GBMLGG |
| TCGA-14-1823-01A | 1 | 1 | 0 | 2 | GBMLGG |
| TCGA-14-1825-01A | 2 | 4 | 1 | 7 | GBMLGG |
| TCGA-14-1829-01A | 2 | 1 | 0 | 3 | GBMLGG |
| TCGA-14-2554-01A | 2 | 4 | 2 | 8 | GBMLGG |
| TCGA-16-1045-01A | 2 | 1 | 3 | 6 | GBMLGG |
| TCGA-19-1390-01A | 5 | 5 | 3 | 13 | GBMLGG |
| TCGA-19-1787-01A | 6 | 3 | 2 | 11 | GBMLGG |
| TCGA-19-2619-01A | 6 | 9 | 7 | 22 | GBMLGG |
| TCGA-19-2620-01A | 6 | 2 | 0 | 8 | GBMLGG |
| TCGA-19-2624-01A | 0 | 2 | 0 | 2 | GBMLGG |
| TCGA-19-2625-01A | 4 | 6 | 2 | 12 | GBMLGG |
| TCGA-19-2629-01A | 9 | 7 | 4 | 20 | GBMLGG |
| TCGA-19-4065-01A | 1 | 5 | 3 | 9 | GBMLGG |
| TCGA-19-5960-01A | 2 | 4 | 5 | 11 | GBMLGG |
| TCGA-26-1442-01A | 4 | 2 | 2 | 8 | GBMLGG |
| TCGA-26-5132-01A | 1 | 3 | 4 | 8 | GBMLGG |
| TCGA-26-5133-01A | 4 | 2 | 10 | 16 | GBMLGG |
| TCGA-26-5134-01A | 1 | 0 | 0 | 1 | GBMLGG |
| TCGA-26-5135-01A | 0 | 0 | 0 | 0 | GBMLGG |
| TCGA-26-5136-01A | 3 | 3 | 2 | 8 | GBMLGG |
| TCGA-26-5139-01A | 0 | 1 | 0 | 1 | GBMLGG |
| TCGA-27-1830-01A | 2 | 2 | 1 | 5 | GBMLGG |
| TCGA-27-1831-01A | 1 | 2 | 2 | 5 | GBMLGG |
| TCGA-27-1832-01A | 4 | 3 | 2 | 9 | GBMLGG |
| TCGA-27-1834-01A | 2 | 0 | 0 | 2 | GBMLGG |
| TCGA-27-1835-01A | 2 | 3 | 3 | 8 | GBMLGG |
| TCGA-27-1837-01A | 1 | 0 | 1 | 2 | GBMLGG |
| TCGA-27-2519-01A | 3 | 1 | 3 | 7 | GBMLGG |
| TCGA-27-2521-01A | 13 | 10 | 7 | 30 | GBMLGG |
| TCGA-27-2523-01A | 6 | 6 | 4 | 16 | GBMLGG |
| TCGA-27-2524-01A | 4 | 6 | 5 | 15 | GBMLGG |
| TCGA-27-2526-01A | 3 | 1 | 3 | 7 | GBMLGG |
| TCGA-27-2528-01A | 3 | 4 | 0 | 7 | GBMLGG |
| TCGA-28-1747-01A | 2 | 3 | 1 | 6 | GBMLGG |
| TCGA-28-1753-01A | 1 | 3 | 0 | 4 | GBMLGG |
| TCGA-28-2509-01A | 0 | 0 | 1 | 1 | GBMLGG |
| TCGA-28-2513-01A | 1 | 5 | 3 | 9 | GBMLGG |
| TCGA-28-2514-01A | 2 | 3 | 2 | 7 | GBMLGG |
| TCGA-28-5204-01A | 5 | 4 | 7 | 16 | GBMLGG |
| TCGA-28-5207-01A | 2 | 4 | 1 | 7 | GBMLGG |
| TCGA-28-5208-01A | 1 | 3 | 2 | 6 | GBMLGG |
| TCGA-28-5209-01A | 2 | 0 | 3 | 5 | GBMLGG |
| TCGA-28-5213-01A | 3 | 3 | 3 | 9 | GBMLGG |
| TCGA-28-5215-01A | 2 | 0 | 1 | 3 | GBMLGG |
| TCGA-28-5216-01A | 0 | 0 | 2 | 2 | GBMLGG |
| TCGA-28-5218-01A | 1 | 0 | 1 | 2 | GBMLGG |
| TCGA-28-5220-01A | 2 | 6 | 3 | 11 | GBMLGG |
| TCGA-32-1970-01A | 5 | 1 | 2 | 8 | GBMLGG |
| TCGA-32-1980-01A | 0 | 0 | 0 | 0 | GBMLGG |
| TCGA-32-1982-01A | 1 | 0 | 0 | 1 | GBMLGG |
| TCGA-32-2615-01A | 1 | 3 | 5 | 9 | GBMLGG |
| TCGA-32-2616-01A | 5 | 1 | 2 | 8 | GBMLGG |
| TCGA-32-2632-01A | 7 | 1 | 1 | 9 | GBMLGG |
| TCGA-32-2634-01A | 3 | 4 | 4 | 11 | GBMLGG |
| TCGA-32-2638-01A | 1 | 1 | 0 | 2 | GBMLGG |
| TCGA-32-4213-01A | 0 | 2 | 3 | 5 | GBMLGG |
| TCGA-32-5222-01A | 2 | 1 | 1 | 4 | GBMLGG |
| TCGA-41-2571-01A | 2 | 5 | 4 | 11 | GBMLGG |
| TCGA-41-2572-01A | 3 | 3 | 5 | 11 | GBMLGG |
| TCGA-41-3915-01A | 2 | 1 | 3 | 6 | GBMLGG |
| TCGA-41-4097-01A | 2 | 2 | 1 | 5 | GBMLGG |
| TCGA-41-5651-01A | 8 | 5 | 3 | 16 | GBMLGG |
| TCGA-76-4925-01A | 8 | 3 | 5 | 16 | GBMLGG |
| TCGA-76-4926-01A | 0 | 0 | 0 | 0 | GBMLGG |
| TCGA-76-4928-01A | 2 | 1 | 2 | 5 | GBMLGG |
| TCGA-76-4929-01A | 1 | 4 | 1 | 6 | GBMLGG |
| TCGA-76-4931-01A | 2 | 0 | 3 | 5 | GBMLGG |
| TCGA-06-0238-01A | 1 | 3 | 1 | 5 | GBMLGG |
| TCGA-06-0125-01A | 1 | 3 | 3 | 7 | GBMLGG |
| TCGA-14-0871-01A | 18 | 9 | 4 | 31 | GBMLGG |
| TCGA-06-0157-01A | 1 | 2 | 0 | 3 | GBMLGG |
| TCGA-12-0821-01A | 2 | 3 | 2 | 7 | GBMLGG |
| TCGA-06-0646-01A | 0 | 1 | 1 | 2 | GBMLGG |
| TCGA-14-0789-01A | 0 | 0 | 0 | 0 | GBMLGG |
| TCGA-14-0817-01A | 1 | 6 | 3 | 10 | GBMLGG |
| TCGA-06-0211-01A | 2 | 0 | 0 | 2 | GBMLGG |
| TCGA-06-0168-01A | 2 | 2 | 7 | 11 | GBMLGG |
| TCGA-06-0130-01A | 3 | 3 | 3 | 9 | GBMLGG |
| TCGA-06-0750-01A | 2 | 1 | 4 | 7 | GBMLGG |
| TCGA-06-0141-01A | 1 | 1 | 0 | 2 | GBMLGG |
| TCGA-12-0616-01A | 5 | 3 | 4 | 12 | GBMLGG |
| TCGA-06-0219-01A | 2 | 8 | 5 | 15 | GBMLGG |
| TCGA-14-0787-01A | 5 | 4 | 4 | 13 | GBMLGG |
| TCGA-02-0047-01A | 3 | 2 | 2 | 7 | GBMLGG |
| TCGA-12-0618-01A | 2 | 2 | 3 | 7 | GBMLGG |
| TCGA-06-0132-01A | 0 | 1 | 2 | 3 | GBMLGG |
| TCGA-15-0742-01A | 4 | 4 | 3 | 11 | GBMLGG |
| TCGA-08-0386-01A | 4 | 7 | 5 | 16 | GBMLGG |
| TCGA-06-0187-01A | 6 | 2 | 3 | 11 | GBMLGG |
| TCGA-16-0846-01A | 5 | 2 | 2 | 9 | GBMLGG |
| TCGA-06-0649-01A | 1 | 4 | 3 | 8 | GBMLGG |
| TCGA-12-0619-01A | 0 | 3 | 2 | 5 | GBMLGG |
| TCGA-06-0744-01A | 5 | 3 | 3 | 11 | GBMLGG |
| TCGA-06-0882-01A | 2 | 3 | 2 | 7 | GBMLGG |
| TCGA-06-0174-01A | 0 | 2 | 0 | 2 | GBMLGG |
| TCGA-06-0210-01A | 3 | 0 | 0 | 3 | GBMLGG |
| TCGA-06-0129-01A | 5 | 5 | 1 | 11 | GBMLGG |
| TCGA-06-0190-01A | 2 | 8 | 4 | 14 | GBMLGG |
| TCGA-06-0158-01A | 2 | 4 | 2 | 8 | GBMLGG |
| TCGA-06-0745-01A | 5 | 10 | 6 | 21 | GBMLGG |
| TCGA-06-0645-01A | 1 | 3 | 3 | 7 | GBMLGG |
| TCGA-06-0686-01A | 0 | 4 | 0 | 4 | GBMLGG |
| TCGA-06-0743-01A | 1 | 3 | 0 | 4 | GBMLGG |
| TCGA-14-0790-01A | 0 | 6 | 3 | 9 | GBMLGG |
| TCGA-06-0878-01A | 3 | 2 | 3 | 8 | GBMLGG |
| TCGA-02-0055-01A | 0 | 2 | 1 | 3 | GBMLGG |
| TCGA-06-0184-01A | 3 | 3 | 1 | 7 | GBMLGG |
| TCGA-06-0747-01A | 3 | 4 | 5 | 12 | GBMLGG |
| TCGA-06-0644-01A | 1 | 2 | 1 | 4 | GBMLGG |
| TCGA-06-0749-01A | 2 | 3 | 4 | 9 | GBMLGG |
| TCGA-14-0781-01A | 0 | 1 | 0 | 1 | GBMLGG |
| TCGA-CS-4938-01A | 3 | 1 | 1 | 5 | GBMLGG |
| TCGA-CS-4941-01A | 0 | 1 | 1 | 2 | GBMLGG |
| TCGA-CS-4942-01A | 3 | 2 | 1 | 6 | GBMLGG |
| TCGA-CS-4943-01A | 12 | 9 | 3 | 24 | GBMLGG |
| TCGA-CS-4944-01A | 2 | 3 | 2 | 7 | GBMLGG |
| TCGA-CS-5393-01A | 6 | 2 | 0 | 8 | GBMLGG |
| TCGA-CS-5394-01A | 2 | 0 | 1 | 3 | GBMLGG |
| TCGA-CS-5395-01A | 2 | 4 | 3 | 9 | GBMLGG |
| TCGA-CS-5396-01A | 1 | 0 | 2 | 3 | GBMLGG |
| TCGA-CS-5397-01A | 4 | 3 | 3 | 10 | GBMLGG |
| TCGA-CS-6186-01A | 2 | 1 | 2 | 5 | GBMLGG |
| TCGA-CS-6188-01A | 5 | 4 | 2 | 11 | GBMLGG |
| TCGA-CS-6290-01A | 2 | 1 | 0 | 3 | GBMLGG |
| TCGA-CS-6665-01A | 8 | 5 | 5 | 18 | GBMLGG |
| TCGA-CS-6666-01A | 14 | 11 | 3 | 28 | GBMLGG |
| TCGA-CS-6667-01A | 1 | 0 | 0 | 1 | GBMLGG |
| TCGA-CS-6668-01A | 4 | 1 | 3 | 8 | GBMLGG |
| TCGA-CS-6670-01A | 2 | 0 | 2 | 4 | GBMLGG |
| TCGA-DB-5270-01A | 2 | 0 | 0 | 2 | GBMLGG |
| TCGA-DB-5273-01A | 4 | 0 | 1 | 5 | GBMLGG |
| TCGA-DB-5274-01A | 2 | 0 | 2 | 4 | GBMLGG |
| TCGA-DB-5275-01A | 5 | 5 | 6 | 16 | GBMLGG |
| TCGA-DB-5276-01A | 1 | 0 | 0 | 1 | GBMLGG |
| TCGA-DB-5277-01A | 11 | 15 | 13 | 39 | GBMLGG |
| TCGA-DB-5278-01A | 2 | 0 | 2 | 4 | GBMLGG |
| TCGA-DB-5279-01A | 2 | 0 | 2 | 4 | GBMLGG |
| TCGA-DB-5280-01A | 1 | 1 | 1 | 3 | GBMLGG |
| TCGA-DB-5281-01A | 9 | 8 | 5 | 22 | GBMLGG |
| TCGA-DB-A4X9-01A | 2 | 1 | 1 | 4 | GBMLGG |
| TCGA-DB-A4XA-01A | 2 | 0 | 2 | 4 | GBMLGG |
| TCGA-DB-A4XB-01A | 7 | 5 | 3 | 15 | GBMLGG |
| TCGA-DB-A4XC-01A | 5 | 3 | 2 | 10 | GBMLGG |
| TCGA-DB-A4XD-01A | 2 | 1 | 1 | 4 | GBMLGG |
| TCGA-DB-A4XE-01A | 2 | 0 | 0 | 2 | GBMLGG |
| TCGA-DB-A4XF-01A | 3 | 4 | 3 | 10 | GBMLGG |
| TCGA-DB-A4XG-01A | 4 | 2 | 4 | 10 | GBMLGG |
| TCGA-DB-A4XH-01A | 1 | 1 | 2 | 4 | GBMLGG |
| TCGA-DB-A64L-01A | 6 | 2 | 3 | 11 | GBMLGG |
| TCGA-DB-A64O-01A | 1 | 0 | 0 | 1 | GBMLGG |
| TCGA-DB-A64P-01A | 4 | 2 | 3 | 9 | GBMLGG |
| TCGA-DB-A64Q-01A | 5 | 2 | 2 | 9 | GBMLGG |
| TCGA-DB-A64R-01A | 1 | 1 | 2 | 4 | GBMLGG |
| TCGA-DB-A64S-01A | 0 | 0 | 0 | 0 | GBMLGG |
| TCGA-DB-A64U-01A | 3 | 0 | 3 | 6 | GBMLGG |
| TCGA-DB-A64V-01A | 3 | 2 | 2 | 7 | GBMLGG |
| TCGA-DB-A64W-01A | 5 | 3 | 6 | 14 | GBMLGG |
| TCGA-DB-A64X-01A | 4 | 6 | 1 | 11 | GBMLGG |
| TCGA-DB-A75K-01A | 1 | 3 | 3 | 7 | GBMLGG |
| TCGA-DB-A75L-01A | 9 | 4 | 4 | 17 | GBMLGG |
| TCGA-DB-A75M-01A | 2 | 0 | 1 | 3 | GBMLGG |
| TCGA-DB-A75O-01A | 6 | 3 | 1 | 10 | GBMLGG |
| TCGA-DB-A75P-01A | 0 | 0 | 0 | 0 | GBMLGG |
| TCGA-DH-5140-01A | 2 | 0 | 1 | 3 | GBMLGG |
| TCGA-DH-5141-01A | 2 | 0 | 2 | 4 | GBMLGG |
| TCGA-DH-5142-01A | 4 | 3 | 2 | 9 | GBMLGG |
| TCGA-DH-5143-01A | 0 | 0 | 0 | 0 | GBMLGG |
| TCGA-DH-5144-01A | 3 | 1 | 3 | 7 | GBMLGG |
| TCGA-DH-A669-01A | 6 | 1 | 4 | 11 | GBMLGG |
| TCGA-DH-A66B-01A | 15 | 6 | 13 | 34 | GBMLGG |
| TCGA-DH-A66D-01A | 5 | 5 | 1 | 11 | GBMLGG |
| TCGA-DH-A66F-01A | 1 | 1 | 2 | 4 | GBMLGG |
| TCGA-DH-A66G-01A | 6 | 5 | 2 | 13 | GBMLGG |
| TCGA-DH-A7UR-01A | 3 | 2 | 3 | 8 | GBMLGG |
| TCGA-DH-A7US-01A | 2 | 0 | 2 | 4 | GBMLGG |
| TCGA-DH-A7UT-01A | 4 | 1 | 4 | 9 | GBMLGG |
| TCGA-DH-A7UU-01A | 7 | 2 | 5 | 14 | GBMLGG |
| TCGA-DH-A7UV-01A | 5 | 2 | 2 | 9 | GBMLGG |
| TCGA-DU-5847-01A | 5 | 3 | 3 | 11 | GBMLGG |
| TCGA-DU-5849-01A | 5 | 3 | 4 | 12 | GBMLGG |
| TCGA-DU-5851-01A | 5 | 4 | 3 | 12 | GBMLGG |
| TCGA-DU-5852-01A | 3 | 3 | 1 | 7 | GBMLGG |
| TCGA-DU-5853-01A | 2 | 0 | 0 | 2 | GBMLGG |
| TCGA-DU-5854-01A | 2 | 2 | 2 | 6 | GBMLGG |
| TCGA-DU-5855-01A | 7 | 6 | 4 | 17 | GBMLGG |
| TCGA-DU-5871-01A | 0 | 0 | 0 | 0 | GBMLGG |
| TCGA-DU-5872-01A | 5 | 5 | 4 | 14 | GBMLGG |
| TCGA-DU-5874-01A | 3 | 1 | 3 | 7 | GBMLGG |
| TCGA-DU-6392-01A | 1 | 2 | 0 | 3 | GBMLGG |
| TCGA-DU-6393-01A | 3 | 1 | 3 | 7 | GBMLGG |
| TCGA-DU-6394-01A | 3 | 1 | 3 | 7 | GBMLGG |
| TCGA-DU-6395-01A | 5 | 3 | 1 | 9 | GBMLGG |
| TCGA-DU-6396-01A | 13 | 7 | 2 | 22 | GBMLGG |
| TCGA-DU-6397-01A | 4 | 2 | 3 | 9 | GBMLGG |
| TCGA-DU-6399-01A | 6 | 5 | 0 | 11 | GBMLGG |
| TCGA-DU-6400-01A | 2 | 0 | 2 | 4 | GBMLGG |
| TCGA-DU-6401-01A | 3 | 5 | 5 | 13 | GBMLGG |
| TCGA-DU-6402-01A | 5 | 7 | 1 | 13 | GBMLGG |
| TCGA-DU-6403-01A | 5 | 3 | 3 | 11 | GBMLGG |
| TCGA-DU-6404-01A | 1 | 0 | 0 | 1 | GBMLGG |
| TCGA-DU-6405-01A | 3 | 1 | 2 | 6 | GBMLGG |
| TCGA-DU-6406-01A | 0 | 0 | 0 | 0 | GBMLGG |
| TCGA-DU-6407-01A | 5 | 6 | 1 | 12 | GBMLGG |
| TCGA-DU-6408-01A | 5 | 5 | 3 | 13 | GBMLGG |
| TCGA-DU-6410-01A | 4 | 0 | 4 | 8 | GBMLGG |
| TCGA-DU-6542-01A | 6 | 5 | 4 | 15 | GBMLGG |
| TCGA-DU-7006-01A | 3 | 6 | 4 | 13 | GBMLGG |
| TCGA-DU-7007-01A | 7 | 3 | 5 | 15 | GBMLGG |
| TCGA-DU-7008-01A | 3 | 2 | 0 | 5 | GBMLGG |
| TCGA-DU-7009-01A | 2 | 2 | 2 | 6 | GBMLGG |
| TCGA-DU-7010-01A | 11 | 9 | 1 | 21 | GBMLGG |
| TCGA-DU-7011-01A | 0 | 1 | 1 | 2 | GBMLGG |
| TCGA-DU-7012-01A | 4 | 3 | 4 | 11 | GBMLGG |
| TCGA-DU-7013-01A | 2 | 0 | 0 | 2 | GBMLGG |
| TCGA-DU-7015-01A | 7 | 6 | 0 | 13 | GBMLGG |
| TCGA-DU-7018-01A | 3 | 0 | 2 | 5 | GBMLGG |
| TCGA-DU-7019-01A | 5 | 3 | 2 | 10 | GBMLGG |
| TCGA-DU-7290-01A | 1 | 0 | 0 | 1 | GBMLGG |
| TCGA-DU-7292-01A | 3 | 2 | 5 | 10 | GBMLGG |
| TCGA-DU-7294-01A | 4 | 2 | 3 | 9 | GBMLGG |
| TCGA-DU-7298-01A | 9 | 7 | 8 | 24 | GBMLGG |
| TCGA-DU-7299-01A | 4 | 2 | 1 | 7 | GBMLGG |
| TCGA-DU-7300-01A | 1 | 0 | 2 | 3 | GBMLGG |
| TCGA-DU-7301-01A | 4 | 6 | 3 | 13 | GBMLGG |
| TCGA-DU-7302-01A | 5 | 2 | 5 | 12 | GBMLGG |
| TCGA-DU-7304-01A | 4 | 1 | 0 | 5 | GBMLGG |
| TCGA-DU-7306-01A | 7 | 5 | 2 | 14 | GBMLGG |
| TCGA-DU-7309-01A | 3 | 3 | 2 | 8 | GBMLGG |
| TCGA-DU-8158-01A | 0 | 1 | 3 | 4 | GBMLGG |
| TCGA-DU-8161-01A | 4 | 3 | 5 | 12 | GBMLGG |
| TCGA-DU-8162-01A | 1 | 1 | 0 | 2 | GBMLGG |
| TCGA-DU-8163-01A | 0 | 0 | 0 | 0 | GBMLGG |
| TCGA-DU-8164-01A | 2 | 0 | 2 | 4 | GBMLGG |
| TCGA-DU-8165-01A | 2 | 0 | 0 | 2 | GBMLGG |
| TCGA-DU-8166-01A | 1 | 0 | 1 | 2 | GBMLGG |
| TCGA-DU-8167-01A | 6 | 4 | 1 | 11 | GBMLGG |
| TCGA-DU-8168-01A | 2 | 4 | 4 | 10 | GBMLGG |
| TCGA-DU-A5TP-01A | 5 | 4 | 3 | 12 | GBMLGG |
| TCGA-DU-A5TR-01A | 6 | 1 | 3 | 10 | GBMLGG |
| TCGA-DU-A5TS-01A | 7 | 2 | 3 | 12 | GBMLGG |
| TCGA-DU-A5TT-01A | 2 | 3 | 2 | 7 | GBMLGG |
| TCGA-DU-A5TU-01A | 8 | 7 | 6 | 21 | GBMLGG |
| TCGA-DU-A5TW-01A | 5 | 5 | 1 | 11 | GBMLGG |
| TCGA-DU-A5TY-01A | 4 | 4 | 2 | 10 | GBMLGG |
| TCGA-DU-A6S2-01A | 2 | 0 | 2 | 4 | GBMLGG |
| TCGA-DU-A6S3-01A | 3 | 0 | 3 | 6 | GBMLGG |
| TCGA-DU-A6S6-01A | 3 | 1 | 2 | 6 | GBMLGG |
| TCGA-DU-A6S7-01A | 5 | 1 | 1 | 7 | GBMLGG |
| TCGA-DU-A6S8-01A | 3 | 2 | 2 | 7 | GBMLGG |
| TCGA-DU-A76K-01A | 0 | 5 | 3 | 8 | GBMLGG |
| TCGA-DU-A76L-01A | 0 | 1 | 1 | 2 | GBMLGG |
| TCGA-DU-A76O-01A | 4 | 3 | 2 | 9 | GBMLGG |
| TCGA-DU-A76R-01A | 2 | 1 | 5 | 8 | GBMLGG |
| TCGA-DU-A7T6-01A | 2 | 0 | 0 | 2 | GBMLGG |
| TCGA-DU-A7T8-01A | 3 | 2 | 0 | 5 | GBMLGG |
| TCGA-DU-A7TA-01A | 8 | 12 | 4 | 24 | GBMLGG |
| TCGA-DU-A7TB-01A | 2 | 2 | 0 | 4 | GBMLGG |
| TCGA-DU-A7TC-01A | 5 | 5 | 4 | 14 | GBMLGG |
| TCGA-DU-A7TD-01A | 0 | 0 | 0 | 0 | GBMLGG |
| TCGA-DU-A7TG-01A | 1 | 1 | 0 | 2 | GBMLGG |
| TCGA-DU-A7TJ-01A | 6 | 4 | 6 | 16 | GBMLGG |
| TCGA-E1-5302-01A | 3 | 4 | 2 | 9 | GBMLGG |
| TCGA-E1-5303-01A | 5 | 4 | 0 | 9 | GBMLGG |
| TCGA-E1-5304-01A | 15 | 22 | 5 | 42 | GBMLGG |
| TCGA-E1-5305-01A | 6 | 6 | 6 | 18 | GBMLGG |
| TCGA-E1-5307-01A | 10 | 12 | 4 | 26 | GBMLGG |
| TCGA-E1-5311-01A | 2 | 0 | 2 | 4 | GBMLGG |
| TCGA-E1-5318-01A | 2 | 0 | 2 | 4 | GBMLGG |
| TCGA-E1-5319-01A | 1 | 2 | 3 | 6 | GBMLGG |
| TCGA-E1-5322-01A | 5 | 3 | 4 | 12 | GBMLGG |
| TCGA-E1-A7YD-01A | 6 | 3 | 2 | 11 | GBMLGG |
| TCGA-E1-A7YE-01A | 9 | 4 | 6 | 19 | GBMLGG |
| TCGA-E1-A7YH-01A | 3 | 1 | 2 | 6 | GBMLGG |
| TCGA-E1-A7YI-01A | 9 | 7 | 4 | 20 | GBMLGG |
| TCGA-E1-A7YJ-01A | 6 | 1 | 4 | 11 | GBMLGG |
| TCGA-E1-A7YK-01A | 7 | 8 | 0 | 15 | GBMLGG |
| TCGA-E1-A7YL-01A | 2 | 2 | 3 | 7 | GBMLGG |
| TCGA-E1-A7YM-01A | 2 | 5 | 1 | 8 | GBMLGG |
| TCGA-E1-A7YN-01A | 3 | 2 | 3 | 8 | GBMLGG |
| TCGA-E1-A7YO-01A | 2 | 0 | 2 | 4 | GBMLGG |
| TCGA-E1-A7YQ-01A | 2 | 3 | 4 | 9 | GBMLGG |
| TCGA-E1-A7YS-01A | 4 | 0 | 4 | 8 | GBMLGG |
| TCGA-E1-A7YU-01A | 8 | 4 | 3 | 15 | GBMLGG |
| TCGA-E1-A7YV-01A | 8 | 7 | 6 | 21 | GBMLGG |
| TCGA-E1-A7YW-01A | 7 | 2 | 0 | 9 | GBMLGG |
| TCGA-E1-A7YY-01A | 2 | 4 | 3 | 9 | GBMLGG |
| TCGA-E1-A7Z2-01A | 3 | 4 | 7 | 14 | GBMLGG |
| TCGA-E1-A7Z3-01A | 2 | 2 | 1 | 5 | GBMLGG |
| TCGA-E1-A7Z4-01A | 4 | 4 | 3 | 11 | GBMLGG |
| TCGA-E1-A7Z6-01A | 3 | 3 | 2 | 8 | GBMLGG |
| TCGA-EZ-7264-01A | 4 | 1 | 4 | 9 | GBMLGG |
| TCGA-F6-A8O3-01A | 2 | 0 | 2 | 4 | GBMLGG |
| TCGA-F6-A8O4-01A | 4 | 4 | 1 | 9 | GBMLGG |
| TCGA-FG-5962-01A | 2 | 0 | 2 | 4 | GBMLGG |
| TCGA-FG-5963-01A | 9 | 8 | 13 | 30 | GBMLGG |
| TCGA-FG-5964-01A | 4 | 2 | 4 | 10 | GBMLGG |
| TCGA-FG-5965-01A | 7 | 3 | 4 | 14 | GBMLGG |
| TCGA-FG-6688-01A | 1 | 3 | 2 | 6 | GBMLGG |
| TCGA-FG-6689-01A | 3 | 0 | 3 | 6 | GBMLGG |
| TCGA-FG-6690-01A | 2 | 1 | 0 | 3 | GBMLGG |
| TCGA-FG-6691-01A | 1 | 1 | 0 | 2 | GBMLGG |
| TCGA-FG-6692-01A | 2 | 0 | 1 | 3 | GBMLGG |
| TCGA-FG-7634-01A | 1 | 0 | 2 | 3 | GBMLGG |
| TCGA-FG-7636-01A | 5 | 1 | 0 | 6 | GBMLGG |
| TCGA-FG-7638-01A | 2 | 0 | 3 | 5 | GBMLGG |
| TCGA-FG-7641-01A | 2 | 0 | 2 | 4 | GBMLGG |
| TCGA-FG-7643-01A | 1 | 1 | 1 | 3 | GBMLGG |
| TCGA-FG-8181-01A | 0 | 0 | 0 | 0 | GBMLGG |
| TCGA-FG-8182-01A | 3 | 2 | 1 | 6 | GBMLGG |
| TCGA-FG-8185-01A | 6 | 3 | 2 | 11 | GBMLGG |
| TCGA-FG-8186-01A | 3 | 1 | 2 | 6 | GBMLGG |
| TCGA-FG-8187-01A | 2 | 0 | 2 | 4 | GBMLGG |
| TCGA-FG-8188-01A | 5 | 5 | 0 | 10 | GBMLGG |
| TCGA-FG-8191-01A | 1 | 1 | 1 | 3 | GBMLGG |
| TCGA-FG-A4MT-01A | 5 | 2 | 4 | 11 | GBMLGG |
| TCGA-FG-A4MU-01A | 0 | 2 | 0 | 2 | GBMLGG |
| TCGA-FG-A4MW-01A | 2 | 2 | 2 | 6 | GBMLGG |
| TCGA-FG-A4MX-01A | 3 | 2 | 3 | 8 | GBMLGG |
| TCGA-FG-A4MY-01A | 10 | 11 | 1 | 22 | GBMLGG |
| TCGA-FG-A60J-01A | 1 | 1 | 0 | 2 | GBMLGG |
| TCGA-FG-A60K-01A | 2 | 0 | 2 | 4 | GBMLGG |
| TCGA-FG-A60L-01A | 5 | 2 | 2 | 9 | GBMLGG |
| TCGA-FG-A6IZ-01A | 2 | 1 | 3 | 6 | GBMLGG |
| TCGA-FG-A6J1-01A | 2 | 0 | 2 | 4 | GBMLGG |
| TCGA-FG-A6J3-01A | 14 | 17 | 3 | 34 | GBMLGG |
| TCGA-FG-A70Y-01A | 2 | 5 | 1 | 8 | GBMLGG |
| TCGA-FG-A70Z-01A | 0 | 1 | 0 | 1 | GBMLGG |
| TCGA-FG-A710-01A | 1 | 1 | 2 | 4 | GBMLGG |
| TCGA-FG-A711-01A | 6 | 5 | 3 | 14 | GBMLGG |
| TCGA-FG-A713-01A | 3 | 1 | 2 | 6 | GBMLGG |
| TCGA-FG-A87N-01A | 3 | 4 | 5 | 12 | GBMLGG |
| TCGA-FG-A87Q-01A | 4 | 4 | 2 | 10 | GBMLGG |
| TCGA-FN-7833-01A | 1 | 1 | 1 | 3 | GBMLGG |
| TCGA-HT-7467-01A | 2 | 0 | 2 | 4 | GBMLGG |
| TCGA-HT-7468-01A | 2 | 0 | 2 | 4 | GBMLGG |
| TCGA-HT-7469-01A | 8 | 6 | 4 | 18 | GBMLGG |
| TCGA-HT-7470-01A | 6 | 8 | 0 | 14 | GBMLGG |
| TCGA-HT-7471-01A | 2 | 0 | 2 | 4 | GBMLGG |
| TCGA-HT-7472-01A | 2 | 2 | 1 | 5 | GBMLGG |
| TCGA-HT-7473-01A | 6 | 3 | 1 | 10 | GBMLGG |
| TCGA-HT-7474-01A | 2 | 2 | 1 | 5 | GBMLGG |
| TCGA-HT-7475-01A | 9 | 7 | 6 | 22 | GBMLGG |
| TCGA-HT-7476-01A | 5 | 2 | 1 | 8 | GBMLGG |
| TCGA-HT-7477-01A | 19 | 12 | 3 | 34 | GBMLGG |
| TCGA-HT-7478-01A | 6 | 3 | 3 | 12 | GBMLGG |
| TCGA-HT-7479-01A | 0 | 0 | 0 | 0 | GBMLGG |
| TCGA-HT-7480-01A | 2 | 0 | 2 | 4 | GBMLGG |
| TCGA-HT-7481-01A | 2 | 0 | 2 | 4 | GBMLGG |
| TCGA-HT-7482-01A | 1 | 1 | 1 | 3 | GBMLGG |
| TCGA-HT-7483-01A | 4 | 3 | 2 | 9 | GBMLGG |
| TCGA-HT-7485-01A | 2 | 1 | 1 | 4 | GBMLGG |
| TCGA-HT-7601-01A | 3 | 2 | 2 | 7 | GBMLGG |
| TCGA-HT-7602-01A | 0 | 0 | 0 | 0 | GBMLGG |
| TCGA-HT-7603-01A | 1 | 4 | 3 | 8 | GBMLGG |
| TCGA-HT-7604-01A | 3 | 4 | 1 | 8 | GBMLGG |
| TCGA-HT-7605-01A | 2 | 0 | 2 | 4 | GBMLGG |
| TCGA-HT-7606-01A | 8 | 6 | 7 | 21 | GBMLGG |
| TCGA-HT-7607-01A | 3 | 1 | 3 | 7 | GBMLGG |
| TCGA-HT-7608-01A | 2 | 0 | 2 | 4 | GBMLGG |
| TCGA-HT-7609-01A | 2 | 1 | 1 | 4 | GBMLGG |
| TCGA-HT-7610-01A | 3 | 2 | 1 | 6 | GBMLGG |
| TCGA-HT-7611-01A | 4 | 4 | 1 | 9 | GBMLGG |
| TCGA-HT-7616-01A | 2 | 1 | 3 | 6 | GBMLGG |
| TCGA-HT-7620-01A | 1 | 0 | 2 | 3 | GBMLGG |
| TCGA-HT-7676-01A | 6 | 3 | 1 | 10 | GBMLGG |
| TCGA-HT-7677-01A | 2 | 1 | 2 | 5 | GBMLGG |
| TCGA-HT-7680-01A | 0 | 0 | 0 | 0 | GBMLGG |
| TCGA-HT-7681-01A | 2 | 0 | 2 | 4 | GBMLGG |
| TCGA-HT-7684-01A | 0 | 0 | 0 | 0 | GBMLGG |
| TCGA-HT-7686-01A | 4 | 2 | 2 | 8 | GBMLGG |
| TCGA-HT-7687-01A | 2 | 0 | 2 | 4 | GBMLGG |
| TCGA-HT-7688-01A | 8 | 6 | 1 | 15 | GBMLGG |
| TCGA-HT-7689-01A | 5 | 1 | 5 | 11 | GBMLGG |
| TCGA-HT-7690-01A | 7 | 6 | 7 | 20 | GBMLGG |
| TCGA-HT-7691-01A | 0 | 0 | 0 | 0 | GBMLGG |
| TCGA-HT-7692-01A | 2 | 0 | 2 | 4 | GBMLGG |
| TCGA-HT-7693-01A | 4 | 2 | 1 | 7 | GBMLGG |
| TCGA-HT-7694-01A | 2 | 0 | 2 | 4 | GBMLGG |
| TCGA-HT-7695-01A | 2 | 0 | 3 | 5 | GBMLGG |
| TCGA-HT-7854-01A | 0 | 0 | 0 | 0 | GBMLGG |
| TCGA-HT-7855-01A | 2 | 1 | 1 | 4 | GBMLGG |
| TCGA-HT-7856-01A | 2 | 2 | 0 | 4 | GBMLGG |
| TCGA-HT-7857-01A | 6 | 10 | 2 | 18 | GBMLGG |
| TCGA-HT-7858-01A | 2 | 2 | 3 | 7 | GBMLGG |
| TCGA-HT-7860-01A | 0 | 1 | 0 | 1 | GBMLGG |
| TCGA-HT-7873-01A | 14 | 8 | 6 | 28 | GBMLGG |
| TCGA-HT-7874-01A | 1 | 1 | 2 | 4 | GBMLGG |
| TCGA-HT-7875-01A | 2 | 2 | 3 | 7 | GBMLGG |
| TCGA-HT-7877-01A | 1 | 1 | 3 | 5 | GBMLGG |
| TCGA-HT-7879-01A | 3 | 5 | 1 | 9 | GBMLGG |
| TCGA-HT-7880-01A | 0 | 0 | 1 | 1 | GBMLGG |
| TCGA-HT-7881-01A | 2 | 0 | 2 | 4 | GBMLGG |
| TCGA-HT-7882-01A | 1 | 2 | 2 | 5 | GBMLGG |
| TCGA-HT-7884-01A | 1 | 3 | 0 | 4 | GBMLGG |
| TCGA-HT-7902-01A | 5 | 4 | 2 | 11 | GBMLGG |
| TCGA-HT-8010-01A | 1 | 1 | 2 | 4 | GBMLGG |
| TCGA-HT-8011-01A | 2 | 6 | 2 | 10 | GBMLGG |
| TCGA-HT-8012-01A | 2 | 0 | 3 | 5 | GBMLGG |
| TCGA-HT-8013-01A | 1 | 1 | 0 | 2 | GBMLGG |
| TCGA-HT-8015-01A | 0 | 0 | 0 | 0 | GBMLGG |
| TCGA-HT-8018-01A | 2 | 2 | 1 | 5 | GBMLGG |
| TCGA-HT-8104-01A | 3 | 4 | 4 | 11 | GBMLGG |
| TCGA-HT-8105-01A | 2 | 0 | 3 | 5 | GBMLGG |
| TCGA-HT-8106-01A | 10 | 9 | 5 | 24 | GBMLGG |
| TCGA-HT-8107-01A | 0 | 0 | 0 | 0 | GBMLGG |
| TCGA-HT-8108-01A | 7 | 5 | 4 | 16 | GBMLGG |
| TCGA-HT-8109-01A | 2 | 2 | 3 | 7 | GBMLGG |
| TCGA-HT-8110-01A | 0 | 2 | 1 | 3 | GBMLGG |
| TCGA-HT-8111-01A | 8 | 2 | 2 | 12 | GBMLGG |
| TCGA-HT-8113-01A | 1 | 1 | 1 | 3 | GBMLGG |
| TCGA-HT-8114-01A | 4 | 2 | 2 | 8 | GBMLGG |
| TCGA-HT-8563-01A | 8 | 6 | 8 | 22 | GBMLGG |
| TCGA-HT-8564-01A | 2 | 1 | 4 | 7 | GBMLGG |
| TCGA-HT-A4DS-01A | 3 | 2 | 2 | 7 | GBMLGG |
| TCGA-HT-A5R5-01A | 5 | 2 | 2 | 9 | GBMLGG |
| TCGA-HT-A5R7-01A | 4 | 1 | 0 | 5 | GBMLGG |
| TCGA-HT-A5R9-01A | 2 | 0 | 2 | 4 | GBMLGG |
| TCGA-HT-A5RA-01A | 3 | 4 | 3 | 10 | GBMLGG |
| TCGA-HT-A5RB-01A | 3 | 1 | 3 | 7 | GBMLGG |
| TCGA-HT-A5RC-01A | 2 | 2 | 2 | 6 | GBMLGG |
| TCGA-HT-A614-01A | 4 | 4 | 2 | 10 | GBMLGG |
| TCGA-HT-A615-01A | 4 | 2 | 3 | 9 | GBMLGG |
| TCGA-HT-A616-01A | 5 | 1 | 2 | 8 | GBMLGG |
| TCGA-HT-A617-01A | 1 | 0 | 0 | 1 | GBMLGG |
| TCGA-HT-A618-01A | 3 | 5 | 2 | 10 | GBMLGG |
| TCGA-HT-A61B-01A | 8 | 5 | 3 | 16 | GBMLGG |
| TCGA-HT-A61C-01A | 3 | 2 | 3 | 8 | GBMLGG |
| TCGA-HT-A74H-01A | 2 | 5 | 0 | 7 | GBMLGG |
| TCGA-HT-A74J-01A | 2 | 1 | 0 | 3 | GBMLGG |
| TCGA-HT-A74K-01A | 3 | 1 | 4 | 8 | GBMLGG |
| TCGA-HT-A74L-01A | 4 | 1 | 2 | 7 | GBMLGG |
| TCGA-HT-A74O-01A | 7 | 4 | 1 | 12 | GBMLGG |
| TCGA-HW-7486-01A | 2 | 0 | 2 | 4 | GBMLGG |
| TCGA-HW-7487-01A | 2 | 0 | 2 | 4 | GBMLGG |
| TCGA-HW-7489-01A | 3 | 6 | 0 | 9 | GBMLGG |
| TCGA-HW-7490-01A | 1 | 0 | 0 | 1 | GBMLGG |
| TCGA-HW-7491-01A | 3 | 0 | 3 | 6 | GBMLGG |
| TCGA-HW-7495-01A | 2 | 1 | 2 | 5 | GBMLGG |
| TCGA-HW-8319-01A | 2 | 2 | 2 | 6 | GBMLGG |
| TCGA-HW-8320-01A | 2 | 5 | 2 | 9 | GBMLGG |
| TCGA-HW-8321-01A | 5 | 3 | 3 | 11 | GBMLGG |
| TCGA-HW-8322-01A | 6 | 4 | 4 | 14 | GBMLGG |
| TCGA-HW-A5KJ-01A | 3 | 2 | 3 | 8 | GBMLGG |
| TCGA-HW-A5KK-01A | 2 | 1 | 3 | 6 | GBMLGG |
| TCGA-HW-A5KL-01A | 1 | 0 | 1 | 2 | GBMLGG |
| TCGA-HW-A5KM-01A | 4 | 4 | 1 | 9 | GBMLGG |
| TCGA-IK-7675-01A | 9 | 11 | 6 | 26 | GBMLGG |
| TCGA-IK-8125-01A | 2 | 0 | 2 | 4 | GBMLGG |
| TCGA-KT-A74X-01A | 1 | 3 | 3 | 7 | GBMLGG |
| TCGA-KT-A7W1-01A | 1 | 1 | 1 | 3 | GBMLGG |
| TCGA-P5-A5EV-01A | 6 | 1 | 5 | 12 | GBMLGG |
| TCGA-P5-A5EW-01A | 2 | 1 | 1 | 4 | GBMLGG |
| TCGA-P5-A5EX-01A | 4 | 2 | 3 | 9 | GBMLGG |
| TCGA-P5-A5EY-01A | 0 | 0 | 0 | 0 | GBMLGG |
| TCGA-P5-A5EZ-01A | 8 | 4 | 5 | 17 | GBMLGG |
| TCGA-P5-A5F0-01A | 2 | 0 | 2 | 4 | GBMLGG |
| TCGA-P5-A5F1-01A | 2 | 1 | 0 | 3 | GBMLGG |
| TCGA-P5-A5F2-01A | 10 | 7 | 5 | 22 | GBMLGG |
| TCGA-P5-A5F4-01A | 8 | 10 | 2 | 20 | GBMLGG |
| TCGA-P5-A5F6-01A | 0 | 1 | 1 | 2 | GBMLGG |
| TCGA-P5-A72U-01A | 2 | 3 | 1 | 6 | GBMLGG |
| TCGA-P5-A72W-01A | 7 | 4 | 1 | 12 | GBMLGG |
| TCGA-P5-A72X-01A | 1 | 0 | 0 | 1 | GBMLGG |
| TCGA-P5-A72Z-01A | 2 | 0 | 2 | 4 | GBMLGG |
| TCGA-P5-A730-01A | 2 | 0 | 2 | 4 | GBMLGG |
| TCGA-P5-A731-01A | 3 | 5 | 4 | 12 | GBMLGG |
| TCGA-P5-A733-01A | 5 | 4 | 2 | 11 | GBMLGG |
| TCGA-P5-A735-01A | 2 | 1 | 0 | 3 | GBMLGG |
| TCGA-P5-A736-01A | 3 | 2 | 0 | 5 | GBMLGG |
| TCGA-P5-A737-01A | 1 | 1 | 2 | 4 | GBMLGG |
| TCGA-P5-A77W-01A | 2 | 2 | 4 | 8 | GBMLGG |
| TCGA-P5-A77X-01A | 2 | 0 | 3 | 5 | GBMLGG |
| TCGA-P5-A780-01A | 9 | 6 | 1 | 16 | GBMLGG |
| TCGA-P5-A781-01A | 1 | 3 | 3 | 7 | GBMLGG |
| TCGA-QH-A65R-01A | 2 | 0 | 2 | 4 | GBMLGG |
| TCGA-QH-A65S-01A | 8 | 1 | 3 | 12 | GBMLGG |
| TCGA-QH-A65V-01A | 2 | 0 | 2 | 4 | GBMLGG |
| TCGA-QH-A65X-01A | 1 | 1 | 3 | 5 | GBMLGG |
| TCGA-QH-A65Z-01A | 2 | 0 | 3 | 5 | GBMLGG |
| TCGA-QH-A6CS-01A | 2 | 0 | 0 | 2 | GBMLGG |
| TCGA-QH-A6CU-01A | 3 | 2 | 3 | 8 | GBMLGG |
| TCGA-QH-A6CV-01A | 4 | 5 | 0 | 9 | GBMLGG |
| TCGA-QH-A6CW-01A | 4 | 4 | 1 | 9 | GBMLGG |
| TCGA-QH-A6CX-01A | 1 | 1 | 0 | 2 | GBMLGG |
| TCGA-QH-A6CY-01A | 0 | 1 | 2 | 3 | GBMLGG |
| TCGA-QH-A6CZ-01A | 2 | 0 | 2 | 4 | GBMLGG |
| TCGA-QH-A6X3-01A | 4 | 3 | 2 | 9 | GBMLGG |
| TCGA-QH-A6X4-01A | 3 | 2 | 4 | 9 | GBMLGG |
| TCGA-QH-A6X5-01A | 2 | 0 | 2 | 4 | GBMLGG |
| TCGA-QH-A6X8-01A | 2 | 2 | 5 | 9 | GBMLGG |
| TCGA-QH-A6X9-01A | 7 | 7 | 3 | 17 | GBMLGG |
| TCGA-QH-A6XA-01A | 4 | 5 | 1 | 10 | GBMLGG |
| TCGA-QH-A6XC-01A | 2 | 1 | 0 | 3 | GBMLGG |
| TCGA-QH-A86X-01A | 2 | 0 | 2 | 4 | GBMLGG |
| TCGA-QH-A870-01A | 5 | 7 | 4 | 16 | GBMLGG |
| TCGA-R8-A6MK-01A | 2 | 2 | 3 | 7 | GBMLGG |
| TCGA-R8-A6ML-01A | 2 | 0 | 2 | 4 | GBMLGG |
| TCGA-R8-A6MO-01A | 2 | 2 | 2 | 6 | GBMLGG |
| TCGA-RY-A83X-01A | 3 | 1 | 5 | 9 | GBMLGG |
| TCGA-RY-A83Y-01A | 1 | 1 | 3 | 5 | GBMLGG |
| TCGA-RY-A83Z-01A | 11 | 9 | 12 | 32 | GBMLGG |
| TCGA-RY-A840-01A | 4 | 1 | 4 | 9 | GBMLGG |
| TCGA-RY-A843-01A | 0 | 0 | 0 | 0 | GBMLGG |
| TCGA-RY-A845-01A | 1 | 0 | 1 | 2 | GBMLGG |
| TCGA-RY-A847-01A | 2 | 0 | 2 | 4 | GBMLGG |
| TCGA-S9-A6TS-01A | 2 | 1 | 2 | 5 | GBMLGG |
| TCGA-S9-A6TU-01A | 3 | 2 | 1 | 6 | GBMLGG |
| TCGA-S9-A6TV-01A | 5 | 6 | 5 | 16 | GBMLGG |
| TCGA-S9-A6TW-01A | 1 | 2 | 3 | 6 | GBMLGG |
| TCGA-S9-A6TX-01A | 4 | 0 | 4 | 8 | GBMLGG |
| TCGA-S9-A6TY-01A | 3 | 1 | 4 | 8 | GBMLGG |
| TCGA-S9-A6TZ-01A | 4 | 2 | 2 | 8 | GBMLGG |
| TCGA-S9-A6U0-01A | 1 | 2 | 0 | 3 | GBMLGG |
| TCGA-S9-A6U1-01A | 2 | 0 | 1 | 3 | GBMLGG |
| TCGA-S9-A6U2-01A | 2 | 0 | 2 | 4 | GBMLGG |
| TCGA-S9-A6U5-01A | 2 | 0 | 2 | 4 | GBMLGG |
| TCGA-S9-A6U6-01A | 4 | 3 | 1 | 8 | GBMLGG |
| TCGA-S9-A6U8-01A | 1 | 0 | 1 | 2 | GBMLGG |
| TCGA-S9-A6U9-01A | 2 | 0 | 1 | 3 | GBMLGG |
| TCGA-S9-A6UA-01A | 8 | 7 | 3 | 18 | GBMLGG |
| TCGA-S9-A6UB-01A | 4 | 1 | 4 | 9 | GBMLGG |
| TCGA-S9-A6WD-01A | 6 | 4 | 4 | 14 | GBMLGG |
| TCGA-S9-A6WE-01A | 2 | 0 | 2 | 4 | GBMLGG |
| TCGA-S9-A6WG-01A | 6 | 5 | 0 | 11 | GBMLGG |
| TCGA-S9-A6WH-01A | 4 | 0 | 2 | 6 | GBMLGG |
| TCGA-S9-A6WI-01A | 0 | 4 | 3 | 7 | GBMLGG |
| TCGA-S9-A6WL-01A | 3 | 0 | 2 | 5 | GBMLGG |
| TCGA-S9-A6WM-01A | 0 | 0 | 4 | 4 | GBMLGG |
| TCGA-S9-A6WN-01A | 2 | 2 | 2 | 6 | GBMLGG |
| TCGA-S9-A6WO-01A | 5 | 5 | 1 | 11 | GBMLGG |
| TCGA-S9-A6WP-01A | 6 | 4 | 4 | 14 | GBMLGG |
| TCGA-S9-A6WQ-01A | 3 | 2 | 3 | 8 | GBMLGG |
| TCGA-S9-A7IQ-01A | 1 | 1 | 2 | 4 | GBMLGG |
| TCGA-S9-A7IS-01A | 6 | 7 | 7 | 20 | GBMLGG |
| TCGA-S9-A7IX-01A | 1 | 5 | 4 | 10 | GBMLGG |
| TCGA-S9-A7IY-01A | 1 | 0 | 2 | 3 | GBMLGG |
| TCGA-S9-A7IZ-01A | 10 | 7 | 3 | 20 | GBMLGG |
| TCGA-S9-A7J0-01A | 16 | 12 | 7 | 35 | GBMLGG |
| TCGA-S9-A7J1-01A | 2 | 4 | 4 | 10 | GBMLGG |
| TCGA-S9-A7J2-01A | 2 | 0 | 2 | 4 | GBMLGG |
| TCGA-S9-A7J3-01A | 3 | 0 | 3 | 6 | GBMLGG |
| TCGA-S9-A7QW-01A | 6 | 6 | 1 | 13 | GBMLGG |
| TCGA-S9-A7QX-01A | 6 | 6 | 2 | 14 | GBMLGG |
| TCGA-S9-A7QY-01A | 4 | 2 | 4 | 10 | GBMLGG |
| TCGA-S9-A7QZ-01A | 3 | 1 | 2 | 6 | GBMLGG |
| TCGA-S9-A7R1-01A | 7 | 4 | 4 | 15 | GBMLGG |
| TCGA-S9-A7R2-01A | 1 | 4 | 2 | 7 | GBMLGG |
| TCGA-S9-A7R3-01A | 5 | 5 | 2 | 12 | GBMLGG |
| TCGA-S9-A7R4-01A | 2 | 2 | 1 | 5 | GBMLGG |
| TCGA-S9-A7R7-01A | 6 | 8 | 5 | 19 | GBMLGG |
| TCGA-S9-A7R8-01A | 5 | 3 | 4 | 12 | GBMLGG |
| TCGA-S9-A89V-01A | 8 | 6 | 1 | 15 | GBMLGG |
| TCGA-S9-A89Z-01A | 11 | 5 | 4 | 20 | GBMLGG |
| TCGA-TM-A7C3-01A | 1 | 1 | 2 | 4 | GBMLGG |
| TCGA-TM-A7C4-01A | 5 | 4 | 3 | 12 | GBMLGG |
| TCGA-TM-A7C5-01A | 2 | 0 | 3 | 5 | GBMLGG |
| TCGA-TM-A7CA-01A | 4 | 3 | 1 | 8 | GBMLGG |
| TCGA-TM-A7CF-01A | 3 | 5 | 1 | 9 | GBMLGG |
| TCGA-TM-A84B-01A | 4 | 3 | 3 | 10 | GBMLGG |
| TCGA-TM-A84C-01A | 0 | 0 | 0 | 0 | GBMLGG |
| TCGA-TM-A84F-01A | 2 | 2 | 1 | 5 | GBMLGG |
| TCGA-TM-A84G-01A | 1 | 3 | 3 | 7 | GBMLGG |
| TCGA-TM-A84H-01A | 9 | 6 | 4 | 19 | GBMLGG |
| TCGA-TM-A84I-01A | 8 | 4 | 3 | 15 | GBMLGG |
| TCGA-TM-A84J-01A | 1 | 1 | 0 | 2 | GBMLGG |
| TCGA-TM-A84L-01A | 1 | 3 | 2 | 6 | GBMLGG |
| TCGA-TM-A84M-01A | 3 | 1 | 2 | 6 | GBMLGG |
| TCGA-TM-A84O-01A | 2 | 0 | 2 | 4 | GBMLGG |
| TCGA-TM-A84Q-01A | 7 | 5 | 2 | 14 | GBMLGG |
| TCGA-TM-A84R-01A | 2 | 0 | 3 | 5 | GBMLGG |
| TCGA-TM-A84S-01A | 2 | 0 | 3 | 5 | GBMLGG |
| TCGA-TM-A84T-01A | 6 | 3 | 2 | 11 | GBMLGG |
| TCGA-TQ-A7RF-01A | 8 | 6 | 6 | 20 | GBMLGG |
| TCGA-TQ-A7RG-01A | 2 | 0 | 2 | 4 | GBMLGG |
| TCGA-TQ-A7RH-01A | 8 | 8 | 6 | 22 | GBMLGG |
| TCGA-TQ-A7RI-01A | 4 | 0 | 3 | 7 | GBMLGG |
| TCGA-TQ-A7RJ-01A | 4 | 4 | 0 | 8 | GBMLGG |
| TCGA-TQ-A7RK-01A | 8 | 4 | 2 | 14 | GBMLGG |
| TCGA-TQ-A7RM-01A | 18 | 16 | 5 | 39 | GBMLGG |
| TCGA-TQ-A7RN-01A | 2 | 0 | 3 | 5 | GBMLGG |
| TCGA-TQ-A7RO-01A | 4 | 2 | 3 | 9 | GBMLGG |
| TCGA-TQ-A7RP-01A | 2 | 0 | 0 | 2 | GBMLGG |
| TCGA-TQ-A7RQ-01A | 2 | 0 | 2 | 4 | GBMLGG |
| TCGA-TQ-A7RR-01A | 9 | 5 | 2 | 16 | GBMLGG |
| TCGA-TQ-A7RU-01A | 2 | 0 | 2 | 4 | GBMLGG |
| TCGA-TQ-A7RV-01A | 7 | 1 | 1 | 9 | GBMLGG |
| TCGA-TQ-A7RW-01A | 6 | 7 | 7 | 20 | GBMLGG |
| TCGA-TQ-A8XE-01A | 7 | 5 | 6 | 18 | GBMLGG |
| TCGA-VM-A8C8-01A | 7 | 6 | 5 | 18 | GBMLGG |
| TCGA-VM-A8C9-01A | 0 | 0 | 0 | 0 | GBMLGG |
| TCGA-VM-A8CA-01A | 2 | 0 | 2 | 4 | GBMLGG |
| TCGA-VM-A8CB-01A | 1 | 2 | 3 | 6 | GBMLGG |
| TCGA-VM-A8CD-01A | 2 | 1 | 2 | 5 | GBMLGG |
| TCGA-VM-A8CE-01A | 2 | 0 | 2 | 4 | GBMLGG |
| TCGA-VM-A8CF-01A | 6 | 6 | 1 | 13 | GBMLGG |
| TCGA-VM-A8CH-01A | 1 | 0 | 0 | 1 | GBMLGG |
| TCGA-VV-A829-01A | 3 | 0 | 3 | 6 | GBMLGG |
| TCGA-VV-A86M-01A | 4 | 3 | 3 | 10 | GBMLGG |
| TCGA-VW-A7QS-01A | 4 | 3 | 3 | 10 | GBMLGG |
| TCGA-VW-A8FI-01A | 1 | 0 | 2 | 3 | GBMLGG |
| TCGA-W9-A837-01A | 1 | 1 | 2 | 4 | GBMLGG |
| TCGA-WH-A86K-01A | 3 | 1 | 0 | 4 | GBMLGG |
| TCGA-WY-A858-01A | 2 | 0 | 2 | 4 | GBMLGG |
| TCGA-WY-A859-01A | 9 | 4 | 6 | 19 | GBMLGG |
| TCGA-WY-A85A-01A | 0 | 0 | 1 | 1 | GBMLGG |
| TCGA-WY-A85B-01A | 3 | 0 | 1 | 4 | GBMLGG |
| TCGA-WY-A85C-01A | 3 | 4 | 2 | 9 | GBMLGG |
| TCGA-WY-A85D-01A | 5 | 9 | 4 | 18 | GBMLGG |
| TCGA-WY-A85E-01A | 10 | 13 | 6 | 29 | GBMLGG |

Supplementary Table S3: Coeffiences of selected 7 genes for model construction.

| **Gene** | **Coef** |
| --- | --- |
| NKX6.3 | 0.093724102 |
| ZAR1 | -0.062002485 |
| CD70 | -0.039968516 |
| PITX2 | -0.089427995 |
| HOXA3 | 0.040121342 |
| HOXB13 | -0.011540812 |
| HOXC9 | 0.034817367 |
